# Insights into xanthine riboswitch structure and metal ion-mediated ligand recognition

**DOI:** 10.1093/nar/gkab486

**Published:** 2021-06-14

**Authors:** Xiaochen Xu, Michaela Egger, Hao Chen, Karolina Bartosik, Ronald Micura, Aiming Ren

**Affiliations:** Life Sciences Institute, Zhejiang University, Hangzhou, Zhejiang 310058, China; Institute of Organic Chemistry, Center for Molecular Biosciences Innsbruck, University of Innsbruck, Innsbruck 6020, Austria; Life Sciences Institute, Zhejiang University, Hangzhou, Zhejiang 310058, China; Institute of Organic Chemistry, Center for Molecular Biosciences Innsbruck, University of Innsbruck, Innsbruck 6020, Austria; Institute of Organic Chemistry, Center for Molecular Biosciences Innsbruck, University of Innsbruck, Innsbruck 6020, Austria; Life Sciences Institute, Zhejiang University, Hangzhou, Zhejiang 310058, China

## Abstract

Riboswitches are conserved functional domains in mRNA that mostly exist in bacteria. They regulate gene expression in response to varying concentrations of metabolites or metal ions. Recently, the *NMT1* RNA motif has been identified to selectively bind xanthine and uric acid, respectively, both are involved in the metabolic pathway of purine degradation. Here, we report a crystal structure of this RNA bound to xanthine. Overall, the riboswitch exhibits a rod-like, continuously stacked fold composed of three stems and two internal junctions. The binding-pocket is determined by the highly conserved junctional sequence J1 between stem P1 and P2a, and engages a long-distance Watson–Crick base pair to junction J2. Xanthine inserts between a G–U pair from the major groove side and is sandwiched between base triples. Strikingly, a Mg^2+^ ion is inner-sphere coordinated to O6 of xanthine and a non-bridging oxygen of a backbone phosphate. Two further hydrated Mg^2+^ ions participate in extensive interactions between xanthine and the pocket. Our structure model is verified by ligand binding analysis to selected riboswitch mutants using isothermal titration calorimetry, and by fluorescence spectroscopic analysis of RNA folding using 2-aminopurine-modified variants. Together, our study highlights the principles of metal ion-mediated ligand recognition by the xanthine riboswitch.

## INTRODUCTION

Riboswitches are non-coding functional RNA domains that are frequently located in the 5′-end regions of bacterial mRNAs (1–6). They contain two overlapping zones, one forming the aptamer for specific recognition of a small ligand, and the other forming the so-called expression platform which becomes restructured upon ligand binding to trigger ON or OFF for gene expression. In most cases, the expression platform utilizes an interplay between terminator and anti-terminator stems (transcriptional regulation) or between repressor and anti-repressor stems (translational regulation) as signal to induce or shut down the expression of genes coding for proteins involved in the biosynthesis or transport of the cognate ligand. Therefore, riboswitch regulation works as a feed-back mechanism (2,7,8).

The first riboswitches were discovered in 2002 (9–12), since then >45 distinct riboswitch-ligand systems have been identified in all three domains of life (2,13). Some of them are specific for metal ions while most of them specifically recognize metabolites (13). Purines and purine derivatives are one of the most abundant classes of metabolites (14). Purine nucleotides such as adenosine 5′-triphosphate (ATP) and guanosine 5′-triphosphate (GTP) are not only essential precursors of RNA and DNA, but also the crucial carriers for supplying cellular energy in biological systems. Other purine nucleotides such as c-di-GMP, c-di-AMP and c-AMP-GMP serve as important signalling molecules for signal transduction (15). In addition, purines also constitute an integral part of several co-factors, such as nicotinamide adenine dinucleotide (NAD), flavin adenine dinucleotide (FAD), coenzyme A (CoA) and *S*-adenosyl methionine (SAM) that enable specific chemical reactions catalysed by protein enzymes (16,17). The intracellular purine levels are maintained in purine metabolism, and deregulations of these processes can cause severe cellular disorders (14). Several important riboswitches were previously identified to regulate the transport and biosynthesis of purines and purine nucleotides in bacteria, including the guanine riboswitch (18), adenine riboswitch (19), 5-amino-4-imidazole carboxamide riboside 5′-triphosphate (ZTP) riboswitch (20), 2′-deoxyguanosine-5′-monophosphate (dGMP) riboswitch (21,22), phosphoribosylpyrophosphate (PRPP) riboswitch (23) and others (24,25).

Very recently, an orphan RNA motif called *NMT1* present in proteobacteria was identified to bind xanthine (25, 26) which is an oxidization product generated during purine degradation in purine metabolism. The consensus sequence model of the *NMT1* motif was derived from phylogenetic analysis and essentially suggested two stems adjoined to a large junction (26). Almost all nucleotides that reside in this junction are highly conserved. In-line probing experiments revealed that the *NMT1* motif binds xanthine with low micromolar affinity in 1:1 stoichiometry, and alternatively, uric acid with 7-fold lower affinity (26). In addition, it was found that the *NMT1* motif binds 8-azaxanthine, which is known as inhibitor of uric acid/urate oxidase (27).

To reveal the three-dimensional fold of the xanthine riboswitch, we set out for crystallographic experiments. Here, we describe the 2.7 Å resolution structure of *NMT1* RNA in complex with xanthine. The structure reveals a unique RNA architecture adopting a rod-like fold with an intriguingly structured binding pocket that is critically depending on divalent metal ions. Most fascinating is the observation of a Mg^2+^ ion that bridges the xanthine's O6 and a backbone phosphate oxygen atom through inner-sphere coordination. We verified the novel RNA fold by mutational analysis and ligand binding assays based on isothermal titration calorimetry (ITC). Furthermore, insights into the Mg^2+^/xanthine-induced folding path and into local structural dynamics were obtained by fluorescence spectroscopy using synthetic riboswitch variants containing 2-aminopurine fluorophores. On top, we complemented the study with a second structure of the *NMT1* RNA bound to the ligand congener 8-azaxanthine. Our studies highlight the principles underlying RNA-based recognition of xanthine, a key degradation product in purine metabolism.

## MATERIALS AND METHODS

### Preparation of RNA for crystallization

Based on the consensus sequence of *NMT1* RNA motif (26), we introduced GNRA tetra-loop motifs and/or the U1A-protein recognition loop into the variable loop of stem P2b in crystallization (Supplementary Figure S1). The sequence of the *NMT1* riboswitch, followed by the sequence of the self-cleaving HDV ribozyme was cloned into pUT7 plasmids with a T7 RNA polymerase promoter, which was amplified in *E. coli* and linearized by endonuclease Hind III delivering the template for transcription (28). *In vitro* transcription was carried out with T7 RNA polymerase at 37°C, followed by denatured polyacrylamide gel electrophoresis (PAGE) purification. The product RNA was visualized using ultraviolet light at a wavelength of 365 nm, excised and soaked in 0.5× TAE buffer at 4°C. The leach solution was precipitated by the iso-propanol method and washed by 80% ethanol. Then, the lyophilized RNA was dissolved in diethyl pyrocarbonate (DEPC) treated, double-distilled water for next-step experiments.

### Ligands

Xanthine, uric acid, hypoxanthine, adenosine, adenine and inosine were purchased from Yuanye Bio-Technology Co. Ltd. (Shanghai). Xanthine sodium salt and 8-azaxanthine monohydrate were purchased from Sigma-Aldrich.

### Crystallization

Crystals were obtained with a 46 nucleotide long RNA of the sequence 5′-GGAGUAGAAGCGUUCAGCGGCC-GAAA-GGCCGCCCGGAAAUUGCUCC-3′ (*Ideonella* sp. B508-1 (NZ_BADL01000600.1) (Supplementary Figure S1C) in the presence of xanthine: The RNA was diluted to a concentration of 0.4 mM and annealed at 65 °C for 5 min in buffer containing 50 mM HEPES, pH 7.0, 50 mM KCl, 5 mM MgCl_2_ before cooling on ice for half an hour. The ligand xanthine was dissolved in 100 mM sodium hydroxide solution and was added to the RNA solution to reach a final xanthine concentration of 6 mM. The RNA-xanthine complex was incubated on ice for another half an hour, followed by centrifugation at 13 000 rpm for 10 min at 4°C before crystallization. For crystallization, 0.18 μl RNA-ligand complex was mixed with the reservoir solution at an equimolar ratio using the drop vapor diffusion method at 16°C. High-resolution crystals grew from the condition containing 0.1 M MES, pH 6.0, 0.2 M calcium acetate and 10% (v/v) isopropanol after ∼1 week. The crystals were transferred in reservoir solution supplemented with 30% glycerol and flash frozen in liquid nitrogen. For Ir(NH_3_)_6_^3+^ soaking experiments, crystals were transferred in reservoir solution supplemented with 50 mM Ir(NH_3_)_6_^3+^ and allowed to equilibrate for 4 h at 4°C prior to flash freezing. For Mn^2+^ soaking experiments, crystals were transferred in reservoir solution supplemented with 66 mM MnCl_2_ and allowed to equilibrate for 2 h at 4°C prior to flash freezing.

### Structure determination and refinement

All X-ray diffraction data were collected on beam line BL19U1 at the Shanghai Synchrotron Radiation Facility (SSRF) and processed with HKL3000 (HKL Research). The crystals belonged to space group P2_1_2_1_2 and the structure was determined at 2.7 Å resolution. There were two RNA molecules in each asymmetric unit as predicted by Matthews coefficient in CCP4 suite (30). The phase problem was solved with the single-wavelength anomalous diffraction (SAD) method using the anomalous signal collected from the Ir(NH_3_)_6_^3+^-soaked crystals using the AutoSol program in Phenix suite (29) (Supplementary Figure S2). The model was further built in COOT (31) and refined using Refmac5 program in CCP4 (30) and phenix.refine program in Phenix suite (29).

The native and Mn^2+^-soaked crystal structures of the xanthine riboswitch in complex with xanthine, as well as the native structure of the xanthine riboswitch in complex with 8-azaxanthine were determined with the molecular replacement (MR) method using the Phaser MR program in the CCP4 suite (30) (Supplementary Table S1). The Ir(NH_3_)_6_^3+^-soaked structure solved with SAD method was used as the initial model. To minimize the model bias, 5% diffractions were selected randomly as the test set in each structure refinement. The ligand xanthine was added to structure at last step in the model building and refinement. The crystallographic statistics of all X-ray data collection and refinement are listed in Supplementary Table S1.

The two molecules (Mol A and Mol B) in each asymmetric unit stack on each other in an end-to-end way (Supplementary Figure S3A, B). We further note that the junction J1 of each RNA molecule in the asymmetric unit also forms stacking interaction with the stem-loop of P2 from another symmetry-related RNA molecule, in which Mol A contacts Mol B’ and Mol B contacts Mol A’’ (Supplementary Figure S3). Since Mol A had better electron density than Mol B, our structural analysis is predominantly based on the coordinates of Mol A.

### Isothermal titration calorimetry

Isothermal titration calorimetry (ITC) experiments were performed at 25°C on a Microcal PEAQ-ITC microcalorimeter at the National Center for Protein Science·Shanghai (NCPSS). A final concentration of 1.4 mM wild-type and mutant RNAs were refolded at 65°C for 5 min and incubated on ice for an hour after dialysis at 4°C overnight against a buffer containing 50 mM Tris pH 8.0, 50 mM KCl and 10 mM MgCl_2_. To test the impact of MgCl_2_ on binding activity between RNA and xanthine, wild-type RNAs were dialyzed against buffer containing 50 mM Tris pH 8.0, 50 mM KCl, supplemented with different concentration of MgCl_2_ from 0 mM to 20 mM. The sample cell was filled with 200 μl 0.1 mM ligand dissolved in each dialysis buffer. The prepared RNA sample titrated into the ligand solution with an initial 0.4 μl injection, followed by 18 serial 2 μl injections, with 2 min interval between each injection. The reference power was set as 5 μcal s^−1^. Integrated heat data were analyzed via MicroCal PEAQ-ITC Analysis Software provided by the manufacturer using a ‘one set of sites’ binding model. All the binding constants and thermodynamic values are listed in Supplementary Table S2.

### Fluorescence spectroscopy

All steady-state fluorescence spectroscopic experiments were measured on a Cary Eclipse spectrometer (Varian, Australia) equipped with a peltier block, a magnetic stirring device and a RX2000 stopped-flow apparatus (Applied Photophysics Ltd., UK). The data obtained were processed with OriginPro 2018 software (OriginLab, USA). Aminopurine-modified RNA samples were prepared in 0.5 μM concentration in a total volume of 1 ml of buffer (100 mM Tris–HCl, 100 mM KCl, pH 8.4). The samples were heated to 90°C for 2 min, allowed to cool to room temperature, transferred to quartz cuvettes equipped with a small stir bar and held at 20°C in the Peltier controlled sample holder. Then, ligands were manually pipetted in a way not to exceed a total volume increase of 3%. The solution was stirred after ligand addition and allowed to equilibrate for at least 15 min before data collection. Spectra were recorded from 320 to 500 nm using the following instrumental parameters: excitation wavelength, 308 nm; increments, 1 nm; scan rate, 120 nm/min; slit widths, 10 nm. Thermodynamic and kinetic parameters *K*_d_ and *k*_obs_ were obtained as described in reference (44).

## RESULTS AND DISCUSSION

### Design of *NMT1* riboswitch constructs for crystallization and structure solution

**Figure 1.**
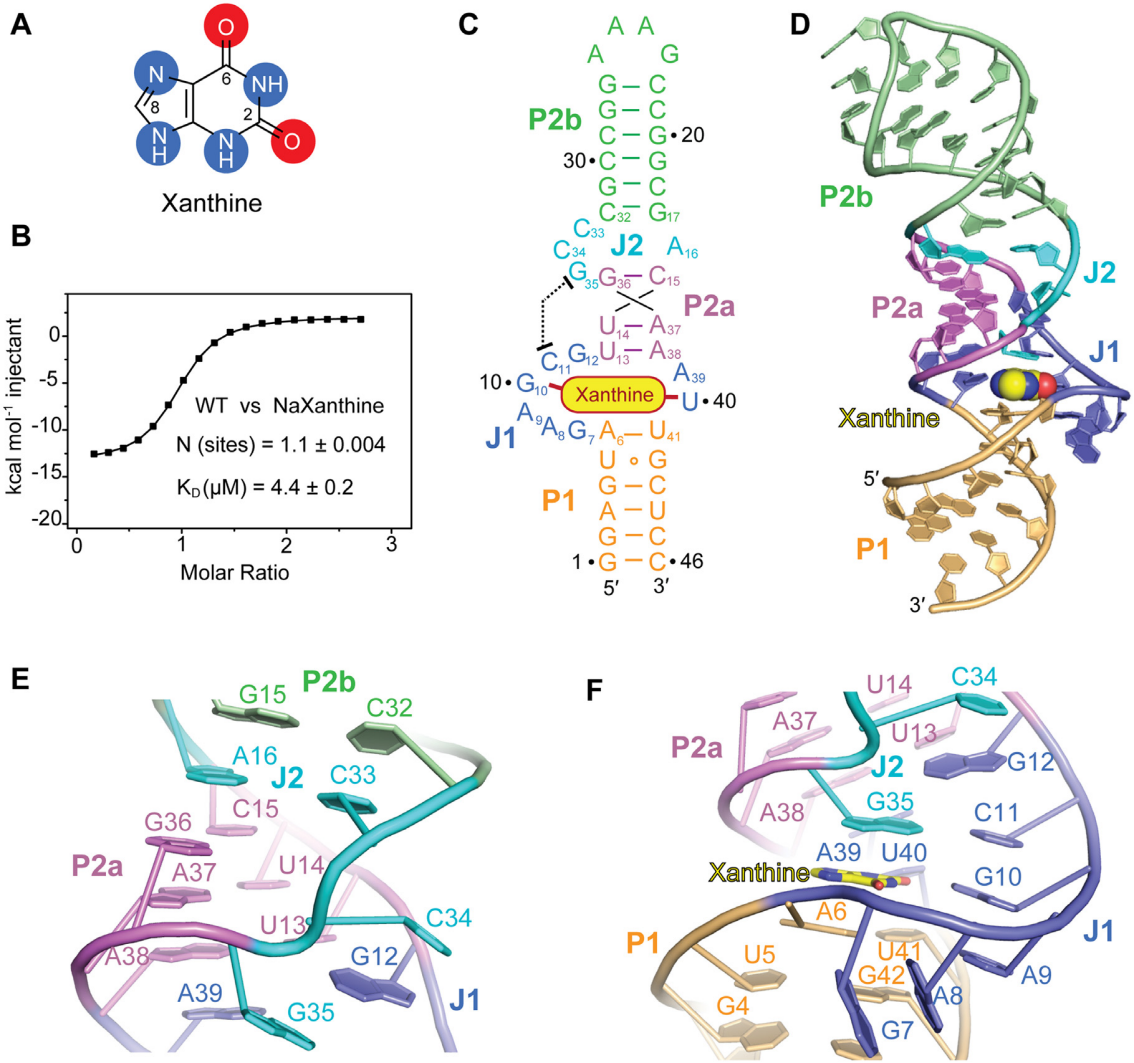
Secondary and tertiary structure of the *NMT1* riboswitch bound to xanthine. (**A**) Chemical structure of xanthine (oxygen atoms are highlighted with red shadows, nitrogen atoms are highlighted with blue shadow). (**B**) Exemplary isothermal titration calorimetry (ITC) experiment of wild-type (WT) *NMT1* riboswitch binding with xanthine. Three replicates are shown in Supplementary Figure S4 and the corresponding thermodynamic parameters are listed in Supplementary Table S2. (**C**) Schematic secondary structure of the folding topology based on the *NMT1* riboswitch crystal structure, same colour code as in the cartoon representation in panel (D) is used. (**D**) Cartoon representation of the tertiary structure of the *NMT1* riboswitch bound to xanthine (shown in spheres). (**E**) Close-up view of the long-distance interaction focusing on junction J2. (**F**) Close-up view of the long-distance interaction focusing on junction J1.

The secondary structure model of the aptamer of the *NMT1* riboswitch conforms to a large junction with two stems (P1 and P2), wherein stem P2 contains a small internal bulge splitting it into two segments (P2a and P2b) (Supplementary Figure S1A) (26). We screened a large number of *in vitro* transcribed RNA constructs for crystallization in the presence of xanthine and obtained crystals for a 46 nucleotide long RNA with the sequence from *Ideonella* species that diffracted to 2.7 Å (Figure 1A, C). In this construct, the variable loop that closes stem P2b was replaced by the extra-stable tetranucleotide loop GAAA to facilitate crystallization (Supplementary Figure S1B, C).

We solved the structure with single-wavelength anomalous diffraction (SAD) phasing by collecting the anomalous signal from Ir(NH_3_)_6_^3+^-soaked crystals (Supplementary Figure S2). The X-ray crystallographic statistics are provided in Supplementary Table S1.

The binding affinity of the 46 nt RNA (GAAA loop) to xanthine was determined by isothermal titration calorimetry (ITC) and gave a dissociation constant of *K*_d_ = 4.4 ± 0.2 μM in aqueous buffer containing 10 mM Mg^2+^ cations (Figure 1B, Supplementary Figure S4 and Supplementary Table S2), which was comparable to the binding affinity of the original sequence (GCCC loop) of the xanthine riboswitch (*K*_d_ = 3.6 ± 0.3 μM, Supplementary Figure S1B, S4 and Supplementary Table S2). The stoichiometry of binding approached 1:1, with estimated thermodynamic parameters of Δ*H* = –15.1 ± 0.1 kcal mol^–1^ and Δ*S* = 0.026 kcal mol^–1^K^–1^ (Figure 1B, Supplementary Figure S4 and Supplementary Table S2).

### Overall tertiary fold of the *NMT1* riboswitch in complex with xanthine

The tertiary structure of the *NMT1* xanthine riboswitch is schematically shown in Figure 1C and in cartoon representation in Figure 1D. Three stems named P1 (in orange), P2a (in violet) and P2b (in green) are formed, which is consistent with the predicted secondary structure model for the *NMT1* motif. The stems are connected by a large junction J1 (in blue) and a small bulge J2 (in cyan) (Figure 1C,D and Supplementary Figure S1A). The RNA folds in a rod-like compact helical scaffold (‘I-shape’), in which the three stems exhibit co-axial stacking mediated by the junctional segments (Figure 1C,D). The ligand xanthine is intercalating and becomes an integrated part of the continuous base staple (Figure 1C,D). A view on the overall base stacking alignment of ligand-bound *NMT1* RNA is highlighted in Supplementary Figure S5A. Notably, stem P1 is extended by two base pairs from the originally assigned junctional region J1 by forming one wobble base pair (U5–G42) and one canonical base pair (A6-U41) (Figure 1C, D and Supplementary Figure S1C).

Between stem P2a and P2b, a non-canonical base pair A16-C33 from bulge J2 is formed and interconnects the terminal base pair G36–C15 of stem P2a and the terminal base pair C32–G17 of stem P2b, therefore a continuous helical structure is generated from stem P2a to stem P2b (Figure 1C–E and Supplementary Figure S5A, B). Consequently, the two nucleotides C34 and G35 of J2 are pushed aside from the main helix axis and fold in parallel to stem P2a. Thereby, C34 and G35 reach out to the north terminal of J1 (Figure 1E) and form long-distance interactions with the nucleotides therein (Figure 1C–E). More precisely, C34 and G35 sandwich G12, the north 1st nucleotide from J1 (Figure 1E), and additionally, G35 forms a canonical Watson–Crick base pair with C11, the north 2nd nucleotide from J1 (Figure 1F). C34, G12, C11–G35 and the four-nucleotide line G10–A9–A8–G7 from J1 align continuously stacked, thereby generating the cavity for the ligand (Figure 1F). A39 and U40 located in the opposite strand of J1 also form consecutive stacking interactions between the north terminal base pair A6–U41 of stem P1 and the south terminal base pair U13–A38 of stem P2a (Figure 1F and Supplementary Figure S6C,D), and hence, they further stabilize the strict coaxial alignment of stem P2a, P1 and junction J1 (Supplementary Figure S5C).

Xanthine is bound at the intersection of J1, J2 and stem P1, where it is bracketed by the two strands of J1 and sandwiched by the terminal base pair A6-U41 of stem P1 and the base tiers formed with the nucleotides from J1 and J2 (Figure 1C, D and Supplementary Figure S1A, S5C–E). The final 2*F*_o_*– F*_c_ map of the native xanthine riboswitch structure in complex with xanthine is shown in Supplementary Figure S6 with emphasis on the J1–J2 junctional region (Supplementary Figure S6A) and the binding pocket (Supplementary Figure S6B). We point out that the highly conserved nucleotides (shown in red in Supplementary Figure S1B) are brought into close proximity around the bound xanthine. Their pairing and stacking interactions define the overall tertiary structure of *NMT1* riboswitch and shape the binding pocket specific for xanthine (Supplementary Figures S1A and S7).

### Structural organization of the junctional regions

**Figure 2.**
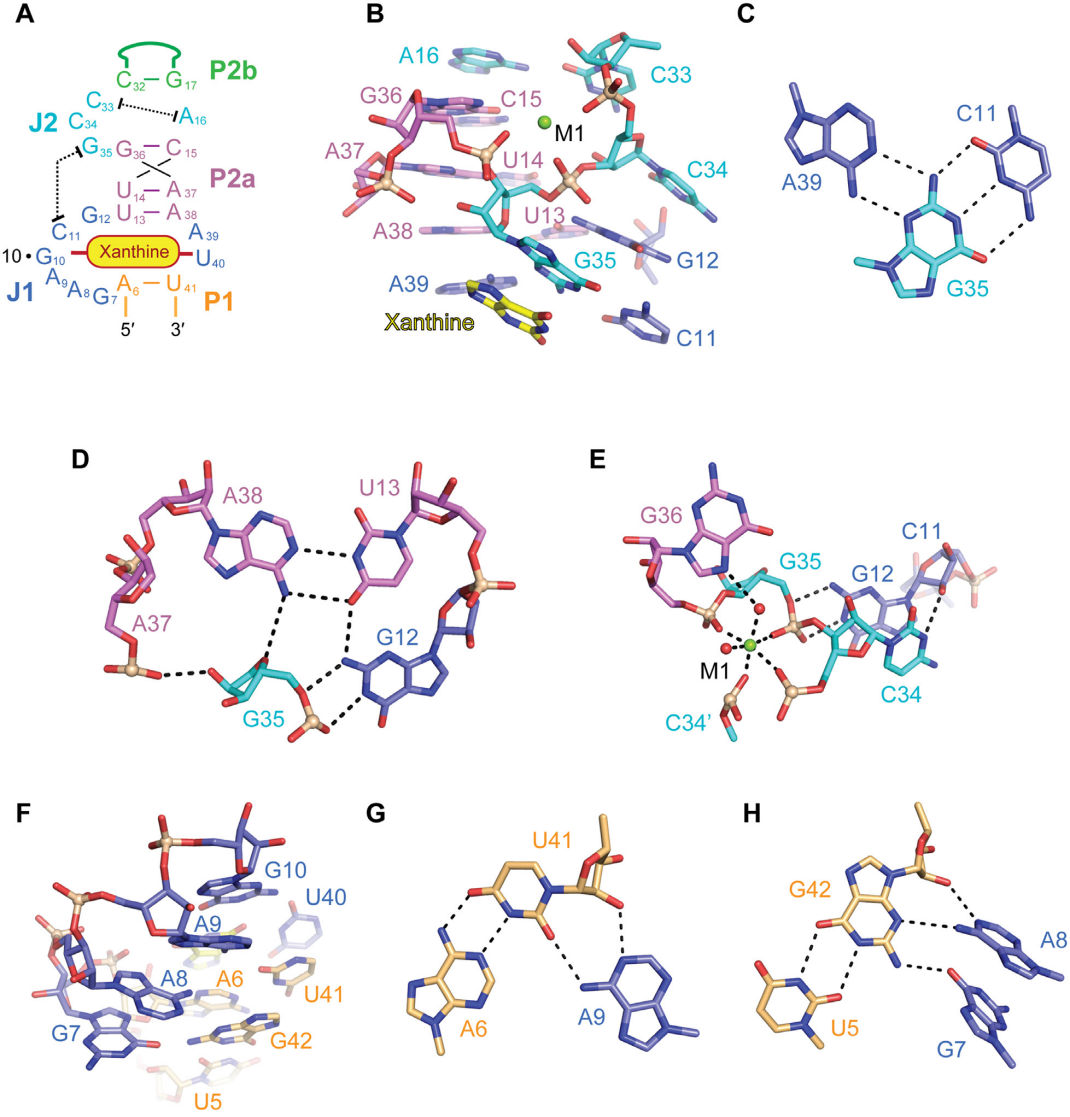
Long-distance interactions between J1 and J2 in the *NMT1* riboswitch. (**A**) Schematic secondary structure of *NMT1* RNA core. (**B**) Structural details of the J1–J2 long-distance interaction shown in sticks representation. A16 (J2) and C33 (J2) form a wobble base pair within J2, while G35 (J2) and C34 (J2) stretch out to form the long-distance interaction with J1. The metal ion M1 (Mg^2+^) (shown as green ball) stabilizes the conformational turn of the J2 chain C33-C34-G35. (**C**) Base triple formed by C11 (J1), G35 (J2) and A39 (J1), with C11 (J1) and G35 (J2) Watson-Crick paired and its minor-groove reccognizing the Watson–Crick edge of A39 (J1). (**D**) The Watson–Crick edge of G12 forms two hydrogen bonds with the phosphate of G35, which together interact with the major groove side of A38-U13 (the terminal base pair of stem P2a). Besides, the 2′-OH of G35 also forms one hydrogen bond with the phosphate of A37. (**E**) The metal ion M1 forms inner-sphere coordination with the phosphates of C34, G35, G36 and a symmetry-related C34′, and outer-sphere coordination with N7 of G36. G12 adopts *2*′-*endo* ribose conformation and the 2′-OH forms one hydrogen bond with N3 of C34. (**F**) G7, A8, A9 and G10 continuously stack and interact with the minor groove side of stem P1. (**G**) A9 forms A-minor interaction with A6-U41 (the first north terminal base pair of stem P1). (**H**) G7 and A8 hydrogen bond with U5-G42 (the second north terminal base pair of stem P1).

The tertiary structure of the xanthine riboswitch is crucially determined by the structural organization of junctions J1 and J2. In an expanded view, Figure 2A shows the secondary structure of this region in the riboswitch center, with all nucleotides numbered and their tertiary interactions highlighted. One of the most impressive features of the structure is the formation of long-distance interactions between J1 and J2, which are separated by stem P2a in the secondary structure (Figures 1B–E, 2A and Supplementary Figure S1).

Four nucleotides A16, C33, C34 and G35 constitute junction J2, in which A16 and C33 form a non-cognate base pair with only one H-bond. This pair is tightly stacked between stems P2a and P2b, while C34 and G35 point outwards to reach out for the long-range interaction (Figures 1B-E, 2A and Supplementary Figure S5C). G35 (J2) forms a canonical Watson-Crick base pair with C11 (J1) (Figure 1F). C34 (J2) and the base pair G35-C11 sandwich the base of G12 (J1) and stack on it from both sides (Figure 1F). Further detailed inspection revealed that the base of G35 (J2) not only forms Watson–Crick pairing interaction with C11 (J1), but also pairs with the Watson–Crick edge of A39 (J1) using the sugar edge, thereby a base triple A39 (J1)–G35 (J2)–C11 (J1) is formed as the first tier stacking above the bound xanthine (Figure 2B,C). The main chain (ribose and backbone) of G35 is positioned in the intersection of J1 and stem P2a and forms additional hydrogen bonding interaction with nucleotides from J1 and stem P2a (Figure 2B–E). The ribose of G35 (J2) forms one hydrogen bond with the 6-NH2 of A38 and an additional one with the phosphate oxygen of A37, while the phosphate oxygen of G35 (J2) forms two hydrogen bonds with the Watson-Crick edge of G12 from J1 (Figure 2D). G12 (J1) is located in the major groove side of the terminal base pair A38–U13 of stem P2a and is further stabilized by one hydrogen bond formed between 2-NH_2_ of G12 (J1) and O4 of U13 (P2a) (Figure 2D). The ribose of G12 (J1) adopts *2*′*-endo* conformation and forms one additional hydrogen bond with N3 of C34 (J2) beside the base stacking interaction between G12 (J1) and C34 (J1) (Figure 2E).

As briefly addressed before, C33 from J2 participates in the long helical base staple, while C34 and G35 are directed outwards to form the long-distance interaction. It is notable that the main chain of C33–C34–G35 (J2) and G36 (P2a) form a three-side rectangle with two successive right angle turns at the sugar positions of C34 and G35 in the bulged region (Figure 2B). One Mg^2+^ cation (M1) is located in the center of the rectangle, which was suggested by the anomalous signal collected from crystals soaked with Mn^2+^ (Supplementary Figure S8). M1 forms inner-sphere coordination with the non-bridging phosphate-oxygen of C34, C34′ (C34 from a symmetric molecule in crystal) G35 and G36, and outer-sphere coordination with N7 of G36 (Figure 2E). With the assistance of M1, the base of G36 from stem P2a appears to be pulled into the main helical scaffold (Figure 2B).

The four consecutive purine nucleotides G10–A9–A8–G7 from J1 employ a successive stacking mode against the minor groove side of stem P1 (Figure 2F). A9 exhibits A-minor interactions with A6-U41, the terminal base pair in stem P1, in which the Watson-Crick edge of this base forms two hydrogen bonds with O2 and 2′-OH of U41 (Figure 2G). A8 stacks on G7 and forms two hydrogen bonds with N3 and 2′-OH of G42, while G7 forms another hydrogen bond with 2-NH_2_ of G42 (Figure 2H). Together, this stacking and base pairing network helps to define the conformation and crosstalk between junction J1 and J2, and hence contributes to the overall stability of the xanthine-bound *NMT1* RNA.

### Pocket architecture and divalent metal ion-mediated binding to xanthine

**Figure 3.**
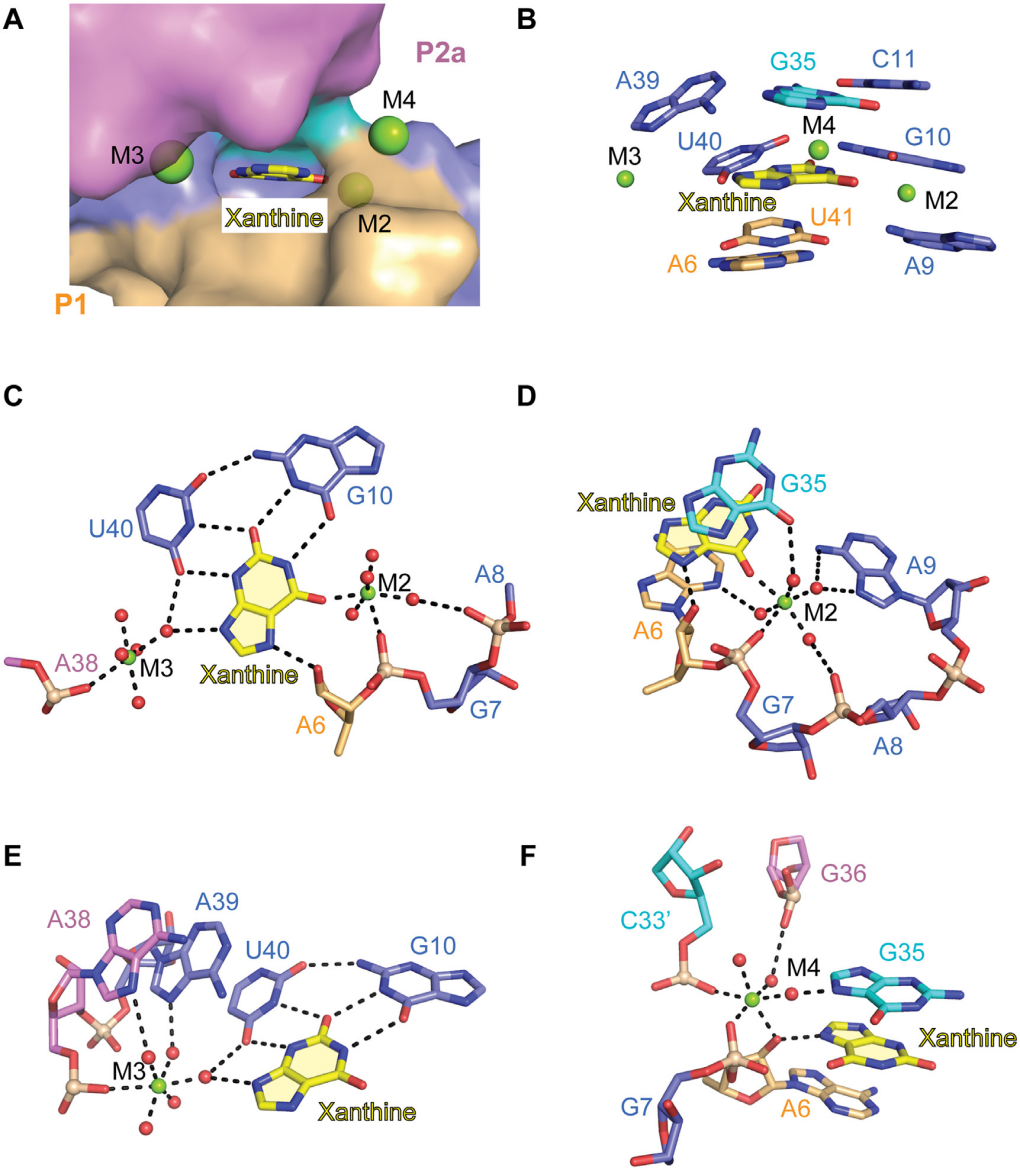
Ligand interaction between xanthine and the *NMT1* riboswitch. (**A**) Xanthine (shown in sticks) is almost completely encapsulated by the RNA binding pocket (shown in surface representation). Three metal ions M2, M3 and M4 are involved in interactions with the ligand. (**B**) Xanthine pairs with G10 and U40, and together are stacked between two base triples, A39–G35–C11 and A9–A6–U41. (**C**) G10 and U40 form one direct hydrogen bond between them and use the rest of their Watson-Crick faces to recognize the Watson–Crick and the minor groove edges of xanthine (i.e. the N3H–C2 = O–N1H urea sub-structure). M2 and M3 are located on opposite sides of xanthine, where M2 forms a direct coordination and M3 forms a water-mediated coordination with xanthine. The 2′-OH of A6 in equatorial position (*2*′*-endo* ribose pucker) forms one hydrogen bond with N7 of xanthine. (**D**) M2 forms direct coordinations with xanthine and the phosphate of G7, while it forms indirect coordinations with the bases of A6, A9 and G35, and the phosphate of A8. Xanthine is sandwiched between A6 and G35. The coordination of M2 with xanthine, G35 and A6 strengthens xanthine binding. (**E**) M3 is directly coordinated to the phosphate of A38, while it forms water-mediated coordination with the bound xanthine, A38, A39 and U40. (**F**) M4 forms direct interactions with the 2′-OH of A6, the phosphates of G7 and a symmetry-related C33′. Additional indirect coordination is observed with the base of G35 and the phosphate of G36. M4 coordinates to both G35 and A6 that stack on the two sides of xanthine.

The surface representation of the xanthine riboswitch ligand-binding pocket makes clear that xanthine (depicted in sticks) intercalates between stem P1 and P2a and becomes almost completely buried with the help of junctional nucleotides (Figure 3A and Supplementary Figure S9A). The bound xanthine is stacked between two base triples, namely A39–G35–C11 (Figures 2C and 3B) and A6–U41–A9 (Figures 2G and 3B). Xanthine itself is also engaged in a triple furnished together with G10 and U40 (Figure 3C). The arrangement is additionally strengthened by the three metal ions M2, M3 and M4 that are located along three sides of the bound xanthine (Figure 3C and Supplementary Figure S9B). The metal ions were assigned based on the 2*F*_o_*– F*_c_ and *F*_o_*– F*_c_ maps guided by the coordination geometries (Supplementary Figure S6C), and the divalent character of the ions was suggested by the anomalous signal collected from the Mn^2+^-soaked crystals (Supplementary Figure S8). All three metal ions are critically involved in the composition of the binding pocket as described below (Figure 3 and Supplementary Figure S9).

Inside the binding pocket, O2 of U40 forms one hydrogen bond with the 2-NH_2_ of G10. Further, both U40 and G10 use their Watson-Crick faces to form two hydrogen bonds with the minor groove edge of xanthine and two hydrogen bonds with the Watson-Crick edge of xanthine respectively (Figure 3C). Therefore, a base triple is formed by clamping the 2-oxo pyrimidine moiety of xanthine (Figure 3C). The final 2*F*_o_*– F*_c_ map of G10, U40 and the bound xanthine is shown in Supplementary Figure S6D. M2 is located in the corner between the Hoogsteen edge and the Watson-Crick edge of xanthine, and importantly, coordinates directly to the O6 atom of xanthine (Figure 3D). Besides, M2 forms an additional inner-sphere coordination with the phosphate of G7 and several outer-sphere coordinations with other residues including the phosphate of A8, the Hoogsteen edge of A9, O6 of G35, and N3 of A6 (Figure 3C, D). It is notable that xanthine intercalates between A6 and G35 with the purine rings stacked in parallel, thus enabling an additional hydrogen bond between 2′-OH of A6 (in *2*′*-endo* ribose conformation) and N7 of xanthine (Figure 3C, D). These interactions are selective for recognizing the Hoogsteen edge of xanthine. M3 is located along the minor groove edge of xanthine and forms inner-sphere coordination with the phosphate of A38 (the terminal residue of stem P2a). It further displays outer-sphere coordination with N7 of A38, N7 of A39, O4 of U40 and N9 of xanthine (Figure 3E). Thereby, the sequence A38–A39–U40 forms a characteristic continuous base staple (Figure 3E and Supplementary Figure S5C). M4 is facing the Hoogsteen edges of G35 and xanthine but is not engaged in direct interactions with xanthine. However, M4 forms inner-sphere coordination to A6 (2′-OH) and outer-sphere coordination to G35 (N7), whose nucleobases sandwich the ligand (Figure 3F). The phosphate of G7 and the phosphate of C33′ from a symmetrical molecule in the crystal both form inner-sphere coordination with M4, while the phosphate of G36 forms outer-sphere coordination. The three metal ion-relying interactions are key for shaping the binding pocket to guarantee high ligand specificity.

Since divalent metal ions are crucially involved in xanthine riboswitch tertiary structure formation, we performed ITC titration experiments at varying Mg^2+^ concentration, ranging from 0 to 20 mM in order to find out the concentrations of divalent cations needed for efficient ligand binding. As shown in Supplementary Figure S10 and S11, binding of xanthine to the *NMT1* RNA requires a Mg^2+^ concentration of at least 0.5 mM, and becomes optimal for Mg^2+^ concentrations around 5 mM or higher.

### Structure-based mutation analysis of the *NMT1* riboswitch

**Figure 4.**
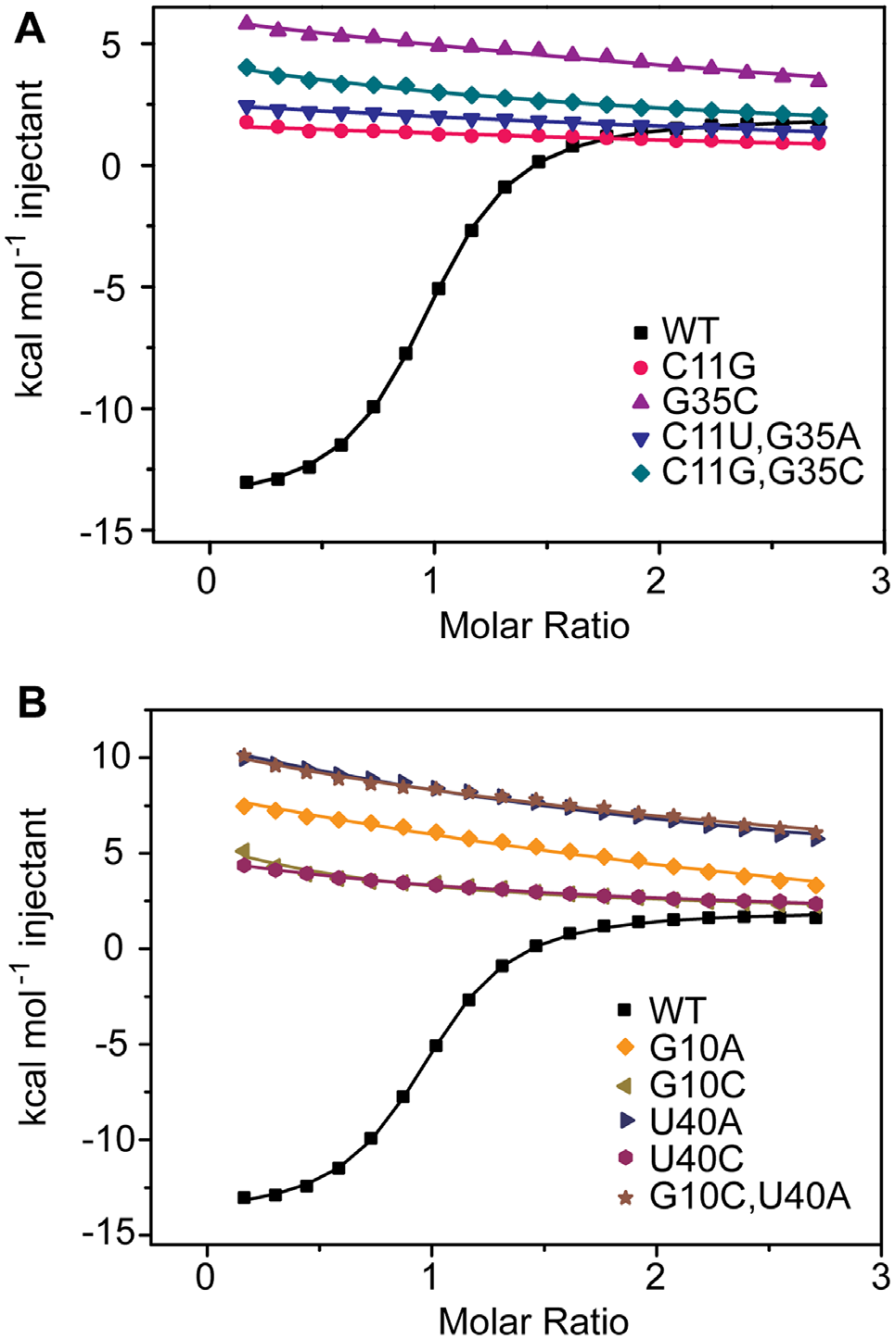
Isothermal titration calorimetry of the *NMT1* riboswitch and structure-based mutants binding with xanthine. (**A**) Overlay of integrated fitted heat plots obtained from ITC experiments of wildtype (WT) in comparison with mutants concerning nucleotides in the long-distance J1/J2 interaction. (**B**) Same as (A) but with mutants concerning nucleotides in the ligand binding site.

The junctional nucleotides are involved in tertiary interactions that appear critical for shaping the binding pocket of the riboswitch (Figure 2A, B). These interactions became visible in the crystal structure and were then evaluated by examining the binding capacity of base mutants of the RNA that had been used for crystallization, applying isothermal titration calorimetry (ITC) as readout (Figure 4 and Supplementary Figure S12).

G35 (J2) forms a long-distance canonical Watson–Crick base pair with C11 (J1) and is additionally hydrogen bonded via its minor groove face to the Watson-Crick face of A39 (J1) (Figure 2C). To assess the impact of these pairing interactions on ligand binding, we made the mutations C11G (J1) and G35C (J2), respectively, to impair their specific interactions. Both mutants resulted in loss of xanthine binding activity (Figure 4A). Then, we prepared constructs with the compensatory mutations C11U–G35A and C11G–G35C, respectively, to retain the possibility for Watson–Crick pairing between nucleotide-11 (J1) and –35 (J2) but to break the hydrogen-bond interactions to A39 (J1) and the divalent metal ion coordination of G35 (J2) (Figure 3C–E); the mutants had no binding activity (Figure 4A). Together, these findings highlight the importance of the base triple A39 (J1)–G35 (J2)–C11 (J1) and conforms with their high conservation in the sequence (26) (Supplementary Figure S1A and B).

G12 residing in J1 is sandwiched between G35 and C34, and additionally forms hydrogen bonds with the phosphate of G35 and the terminal base U13 of stem P2a (Figure 2B and D). Here, mutation of G12 to A resulted in somewhat lower ligand affinity (7-fold decrease), consistent with the loss of a hydrogen bond but retainment of stacking interactions (Supplementary Figure S12A). Additional mutations were tested for C34, which is participating in the J1-J2 long-range interaction by intense base stacking onto G12 and by forming a single hydrogen bond with the 2′-OH of G12 ribose (Figure 2B,E and Supplementary Figure S12B). While the C34A mutant exhibited binding affinity comparable to the wild-type RNA, the C34U and C34G mutants, respectively, resulted in a slight decrease (3- to 4-fold) in binding affinity only (Supplementary Figure S12B). Both G12 and C34 are not highly conserved (Supplementary Figure S1A and B), and this is consistent with our observation that their mutations were largely tolerated.

With respect to junction 2, we tested the internal one-hydrogen bonded A16•C33 pair which stacks on the terminal base pair of stem P2b. While A16 is largely conserved in nucleobase identity, nucleotide-33 is not. The A16G mutant binds xanthine with only 4-fold less affinity, as does the A16G/C33U double mutant (Supplementary Table S2, Supplementary Figure S12C). This is consistent with retained stacking interactions and the continuation of the stem P2b base staple as observed in the WT RNA.

In junction J1, A9 forms an A-minor interaction with the terminal base pair A6-U41 of stem P1 (Figure 2F and G). To test the significance of the A9 base identity for ligand binding, it was mutated to C. Not surprisingly, the A9C mutant did no longer bind to xanthine (Supplementary Figure S12D). As shown in Figure 2H, A8 and G7 form hydrogen bonds with the minor groove side of the second terminal base pair of stem P1, a wobble base pair (U5-G42). When we mutated A8 to C or U individually, both RNAs lost capacity for ligand binding (Supplementary Figure S12D). Additionally, the mutant G7A-A8G was prepared; for the G/A switched RNA, ligand binding was not detectable any more (Figure 2F, H, and Supplementary Figure S12D).

Inside the pocket, G10 and U40 clamp the 2-oxo pyrimidine moiety of xanthine from both sides by forming a base triple (Figure 3C). Individual mutations of these nucleobases (G10A, G10C, U40A, and U40C) were not tolerated (Figure 4B). The double mutant G10C-U40A did neither bind xanthine (Figure 4B). In summary, our ITC-based mutation study is consistent with the three-dimensional structure model of the *NMT1* riboswitch obtained by X-ray crystallography.

### Binding capacity of the *NMT1* riboswitch to xanthine related compounds

**Figure 5.**
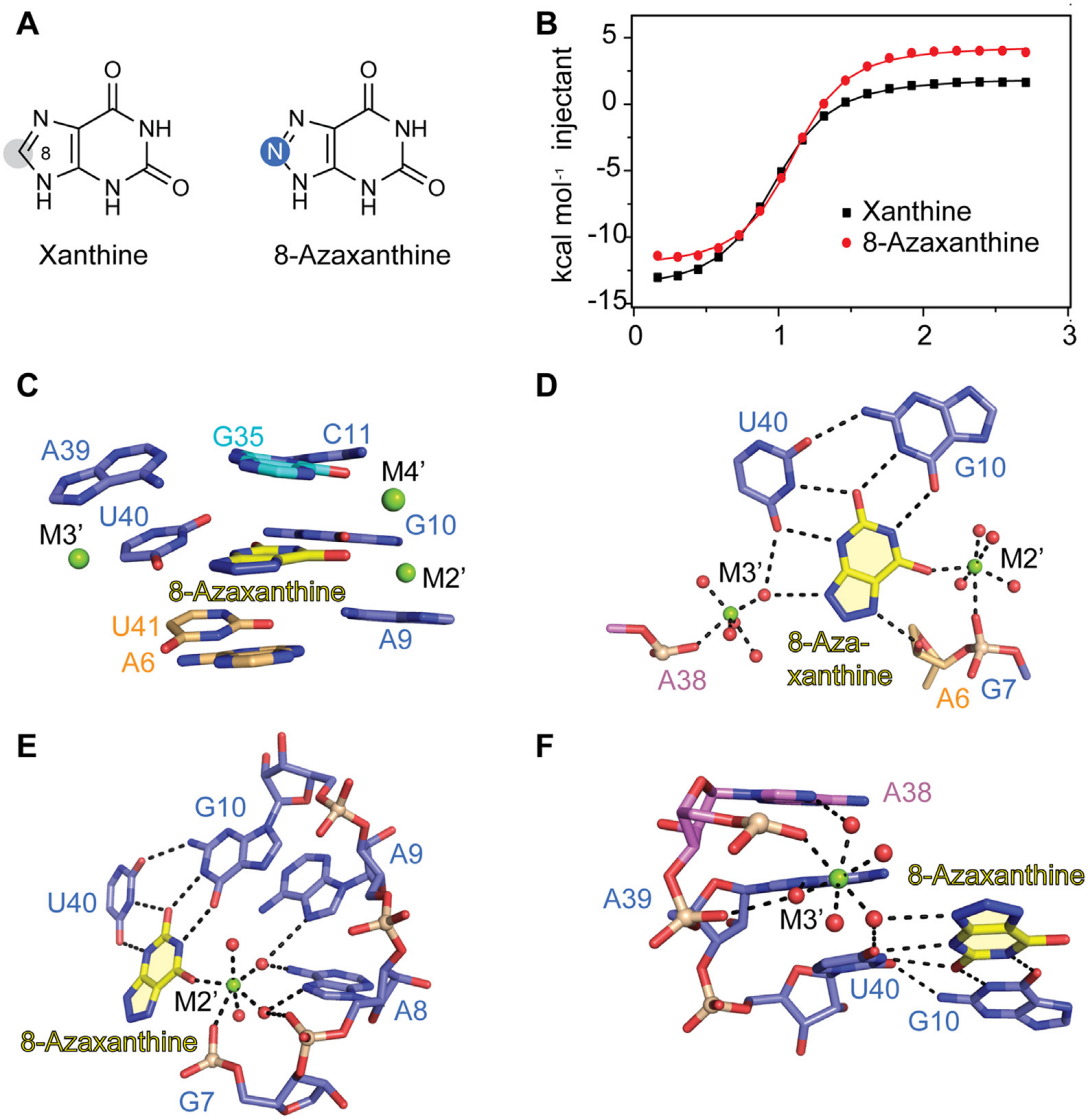
Structure of the *NMT1* riboswitch bound to 8-azaxanthine. (**A**) Chemical structures of xanthine and 8-azaxanthine (different atom identities are highlighted with grey (xanthine) and blue (8-azaxathine) shadows. (**B**) ITC heat plot of the *NMT1* riboswitch binding with xanthine (in black) or 8-azaxathine (in red). (**C**) 8-Azaxathine hydrogen bonds with G10 and U40, and together are stacked between two base triples A39-G35-C11 and A9-A6-U41 as observed in the xanthine-bound structure. Three metal ions M2′, M3′ and M4′ are also retained in the 8-azaxathine-bound structure. (**D**) Hydrogen bonding interactions and metal ion-involved coordination of 8-azaxathine highlighted for the *NMT1* riboswitch pocket. (**E, F**) Metal ion-coordinations of M2′ (E) and M3′ (F) observed in the *NMT1* riboswitch pocket occupied by 8-azaxanthine.

It has been reported in the original study on xanthine riboswitch discovery (26) that 8-azaxanthine and uric acid bind to the *NMT1* motif, while other compounds involved in purine degradation pathways including hypoxanthine, adenine, inosine and adenosine (for chemical structures see Figure 5A and Supplementary Figure S13A) have no or significantly lower binding affinities. Here, we again used isothermal titration calorimetry (ITC) assays to evaluate these ligand-RNA interactions and to confirm the earlier observations from the Breaker lab (Supplementary Figure S13B, S14 and S15). More precisely, 8-azaxanthine gave a similar binding affinity compared to xanthine (*K*_d_ = 3.0 ± 0.1 μM), while uric acid gave an about 7-fold lower binding affinity (*K*_d_ = 20.4 ± 1.7 μM) (Figure 5B, Supplementary Figure S4 and S14, and Supplementary Table S2).

We were successful in co-crystallization and structure determination of the *NMT1* riboswitch in complex with 8-azaxanthine. The overall 3D-folds of the riboswitch bound to xanthine and 8-azaxanthine, respectively, are nearly identical (Figure 1D and Supplementary Figure S16A) as are the recognition modes for xanthine and 8-azaxanthine in the binding pocket (Figure 5C–E). The three characteristic metal ions M2′, M3′ and M4′, assigned to Mg^2+^ according to 2*F*_o_*–* *F*_c_ and *F*_o_*– F*_c_ maps guided by the coordination geometries are present as well (Figure 5D–F and Supplementary Figure S16B) and the interactions of the hydrated metal ions with neighboring nucleotides are also retained for the 8-azaxanthine-riboswitch complex (Figure 3, 5 and Supplementary Figures S9B, S16B).

Both structures reveal that the ligands xanthine and 8-azaxanthine are almost completely encapsulated. The only position that is accessible from the solvent is ligand position C8 (Figure 3C and 5D). Therefore, the replacement of C8-H by a nitrogen atom (as in 8-azaxanthine) or the addition of a carbonyl oxygen at C8 (as in uric acid) is consistent with our observation that 8-azaxanthine and uric acid are well accepted by the riboswitch scaffold (Supplementary Figures S13B and S14). In contrast, the related purines hypoxanthine and adenine do not satisfy the hydrogen donor-acceptor pattern provided by the pocket. In particular, G10 and U40 which are perfectly complementary to the 2-oxo pyrimidine moiety of xanthine (Figure 3C), miss this recognition possibilities if confronted with the pyrimidine moiety of hypoxanthine or adenine, both lacking the 2-oxo group. Hence, the inability to bind any of these compounds is consistent with the H-donor/H-acceptor profile provided in the tight pocket (Supplementary Figures S13 and S15). Furthermore, inosine and adenosine do not bind to the *NMT1* RNA and steric hindrance of the large ribose group at N9 is the likely reason for this observation (Figure 3C, Supplementary Figures S13 and S15).

### Some insights into Mg^2+^ and xanthine dependent riboswitch folding

**Figure 6.**
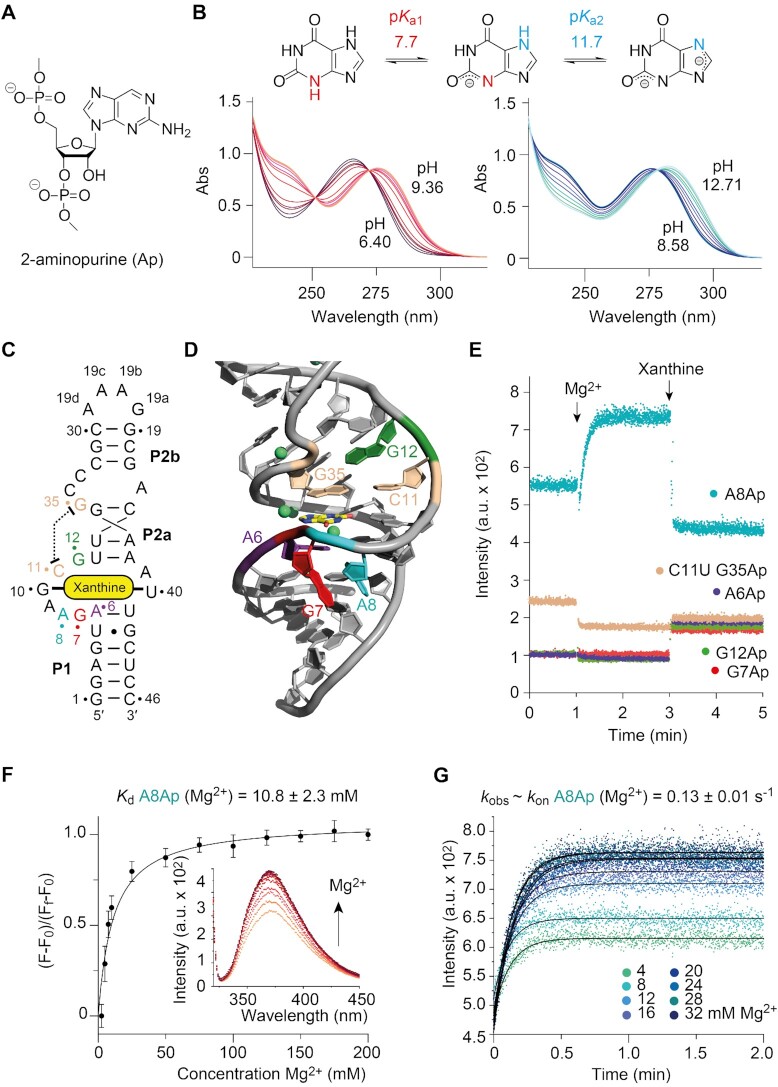
Fluorescence spectroscopic assessment of *NMT1* RNA folding and ligand binding. (**A**) Chemical structure of 2-aminopurine in an RNA chain. (**B**) Protonation equilibria of xanthine (sites of deprotonation are shown according to reference 46) and corresponding UV-spectra obtained by pH titration experiments used for p*K*_a_ determination. (**C**) Secondary structure of *NMT1* RNA with color-coded nucleotides that were individually replaced by Ap. (**D**) Cartoon representation of *NMT1* RNA highlighting Ap substitutions by using the same color code as in (C). (**E**) Real time fluorescence traces for five different Ap riboswitch variants (*c* = 0.5 μM) upon Mg^2+^ (20 mM) and ligand (3 mM) additions; buffer conditions: 100 mM Tris–HCl, pH 8.4, 100 mM KCl. (**F**) Fluorescence changes upon titration of A8Ap RNA (*c* = 0.5 μM) with increasing concentrations of Mg^2+^ ions; normalized fluorescence intensity of the A8Ap variant plotted as a function of Mg^2+^ concentration. The graph shows the best fit of the two parametric quadratic fit. Changes in fluorescence (*F* – *F*_0_) were normalized to the maximum fluorescence measured in saturating concentrations of Mg^2+^ ligand. The obtained *K*_d_ value for Mg^2+^ in 100 mM Tris–HCl, pH 8.4, 100 mM KCl, at 293 K, is indicated; inset: fluorescence emission spectra (λ_ex_ = 308 nm) from 320 to 450 nm of the A8Ap variant for each Mg^2+^ concentration. (**G**) Monitoring the kinetics of Mg^2+^-induced *NMT1* RNA folding using the A8Ap labeled RNA. Exemplary fluorescence traces for Mg^2+^ additions are depicted; conditions: 0.5 μM RNA, 100 mM Tris–HCl, 100 mM KCl, pH 8.4, at 293 K. Final MgCl_2_ concentrations as indicated.

Ligand-induced RNA folding can usually be investigated by applying 2-aminopurine (Ap) modified RNA variants in a fluorescence spectroscopic approach termed 2ApFold (Figure 6A) (8,32,33). One has to be aware that the UV absorbance of xanthine is depending on the pH value of the solution. To underline this, we determined the p*K*_a1_ and p*K*_a2_ values for xanthine (which were 7.7 and 11.7, respectively) by UV-spectroscopic detection of pH titration experiments (Figure 6B). Moreover, because of the spectral overlap of xanthine and 2-aminopurine, limitations are encounter for quantitative 2ApFold measurements. However, on a qualitative level, we were confident that the analysis of riboswitch variants with Ap replacements at selected positions should allow to detect regions of xanthine-induced compaction of the riboswitch fold (Figure 6C, D). Moreover, Mg^2+^-induced local structural rearrangements that take place in the course of RNA pre-folding (in the absence of xanthine) should be detectable, even on a quantitative level.

The xanthine-bound riboswitch fold does not provide any nucleobases that are unstacked and completely exposed to the solvent. Such nucleotide positions are usually used for replacements by Ap, because they are structurally non-invasive and the corresponding RNA variants respond by a pronounced fluorescence increase upon ligand binding. Nevertheless, the xanthine riboswitch fold offers several internal positions where the aminopurine replacement retains the hydrogen bond and/or stacking pattern. These are A8Ap, G7Ap, A6Ap, G12Ap. Moreover, by considering the double mutation C11U-G35Ap, the long-range Watson-Crick base pair that stacks on top of xanthine is retained, and its formation should also be traceable by a putative xanthine-induced fluorescence response. We synthesized all of these variants and the qualitative fluorescence responses of the riboswitch variants (0.5 μM each) upon addition of saturating concentrations of Mg^2+^ ions (20 mM), and subsequently, of xanthine (3 mM) were recorded and are depicted in Figure 6E. Due to solubility of xanthine, we use buffer conditions with a slightly basic pH of 8.4. Interestingly for the majority of Ap variants the Mg^2+^ addition resulted in no (A6Ap, G7Ap, G12Ap) or only a minor (C11U-G35Ap) decrease in fluorescence, suggesting a rather pre-organized RNA fold that largely resembles the final xanthine-bound fold. From this subset, G6Ap, G7Ap and G12Ap experienced a small (onefold) fluorescence increase when xanthine became available, indicating that these nucleobases are slightly more exposed in the ligand-bound fold compared to their positions in the Mg^2+^-induced pre-folded state. Remarkably, the nucleotide at position 8 responded significantly to both ligands and this behavior identifies this region to be the conformationally most flexible one. While during Mg^2+^-triggered preorganization the nucleotide-8 becomes exposed (fluorescence increase) it slides into its finally stacked position between G7 and A9 when xanthine becomes bound (fluorescence decrease). This is consistent with the Mg^2+^ (M2) ‘bridge’ between the backbone of G7-A8 and the O6 of xanthine as seen in the crystal structure.

The pronounced fluorescence increase for Mg^2+^-induced folding of the A8Ap riboswitch variant allowed for quantification. The concentration-dependent fluorescence response data of Mg^2+^ titrations (Figure 6F) were fit using a two-parametric quadratic equation and gave an apparent dissociation constant, *K*_d_ (Mg^2+^) of 10.8 ± 2.3 mM (Figure 6F). Clearly, this *K*_d_ is well above physiologically encountered Mg^2+^ concentrations, however, it is compatible with the assumption that a small population of RNA with exposed A8 is characteristic and likely supportive for ligand recognition in the natural environment (pre-folding). Additionally, we investigated the kinetics of Mg^2+^ binding for this riboswitch region. The rates (*k*_obs_) were determined in Mg^2+^ concentration-dependent manner by fitting the individual fluorescence traces to a single exponential equation, obtained from manually performed measurements (Figure 6G). The rates were in the order of 0.13 s^–1^ and turned out to be independent of Mg^2+^ concentration. This observation indicates that not Mg^2+^ binding itself is rate limiting but rather a change in RNA conformation which is consistent with the conformational rearrangement of A8 as proposed above.

### Concluding remarks

Bacterial RNAs containing the *NMT1* motif turn off gene expression upon ligand sensing, likely by regulating translation initiation (26). Members of this riboswitch class respond to high concentrations of oxidized purines and they regulate genes predominantly associated to purine transport and oxidation, thus avoiding the deleterious effects of accumulation of purine degradation products in the cell (26).

**Figure 7.**
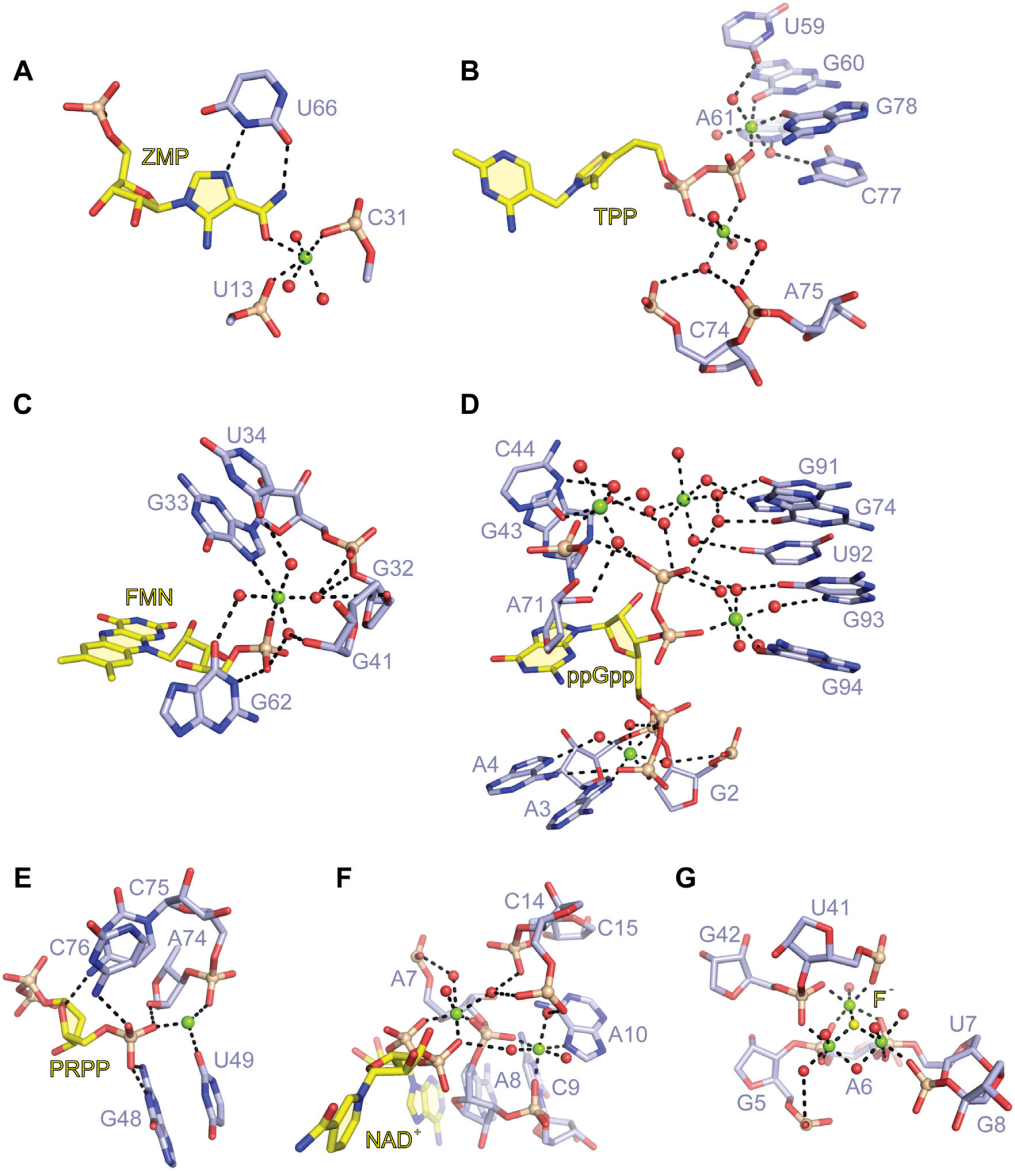
Comparison of Mg^2+^-mediated ligand recognition by various riboswitches. (**A**) The carboxamide oxygen of ZMP applies one inner-sphere coordination with a Mg^2+^ cation in the binding pocket of the ZMP riboswitch (PDB code: 4ZNP). (**B**) The phosphates of TPP form direct coordinations with two Mg^2+^ cations in the binding pocket of the TPP riboswitch (PDB code: 2GDI). (**C**) The phosphate of FMN is recognized by forming a direct and an indirect coordination with the Mg^2+^ cation in the binding pocket of the FMN riboswitch (PDB code: 3F2Q). (**D**) Four Mg^2+^ cations are involved in interactions between ppGpp and the ppGpp riboswitch pocket (PDB code: 6DMC). (**E**) One Mg^2+^ cation mediates the interaction of the phosphate of PRPP and the PRPP riboswitch pocket (PDB code: 6DLT). (**F**) The phosphates of NAD^+^ forms inner-sphere and outer-sphere coordination with two Mg^2+^ cations in the pocket of the NAD-I riboswitch (PDB code: 7D7X). (**G**) Fluoride directly contacts three Mg^2+^ cations, which in turn form direct interactions with five inwardly pointing phosphates of the fluoride riboswitch pocket (PDB code: 4ENC).

There is currently only one other known example of a riboswitch that binds a nucleobase via inner-sphere Mg^2+^ coordination, namely the ZMP (5-amino-4-imidazole carboxamide ribose-5′-monophosphate) riboswitch. This riboswitch is widespread and regulates de novo purine synthesis (20). It recognizes the carboxamide oxygen of ZMP through coordination with a Mg^2+^ cation, which is simultaneous coordinated to two backbone phosphates (34–36) (Figure 7A). More frequently, Mg^2+^-mediated binding of small molecules to RNA concerns the phosphate moieties of these ligands, as observed e.g. for the thiamine pyrophosphate (TPP) riboswitch (37–39), the flavin mononucleotide (FMN) riboswitch (40), guanosine-3′,5′-bispyrophosphate (ppGpp) and α-5′-phosphoribosyl-1′-pyrophosphat (PRPP) riboswitches (41,42), and the nicotinamide adenine dinucleotide (NAD-I) riboswitch (43,44) (Figure 7B–F). The fluoride riboswitch also uses the concept of Mg^2+^ mediated ligand binding. The tiny anion is anchored to the RNA through direct coordination to three Mg^2+^ ions, which in turn are octahedrally coordinated to water molecules and five inwardly pointing backbone phosphates (45) (Figure 7G).

Our structural study reveals the molecular basis for the high specificity of this RNA to bind xanthine and only near cognate ligands such as uric acid and 8-azaxanthine. The key recognition feature is insertion of the xanthine's pyrimidine moiety between an opened G–U base pair, thus enabling shape complementarity and simultaneous recognition of the C2 urea substructure by a maximum of four hydrogen bonds. The 2-oxo group is lacking in hypoxanthine and hence the pocket strongly discriminates against it. The second key feature is Mg^2+^-mediated recognition of the xanthine-O6 atom. This crucial interaction cannot be compensated by the isosteric N6 group of adenine related derivatives that cannot bind. Our structure also explains the similar affinity for 8-azaxanthine and the only slightly lower one for uric acid, because the purine-8 position is solvent-accessible and the only position that is not engaged in the tight hydrogen bonding and stacking network generated by the riboswitch pocket.

**Figure 8.**
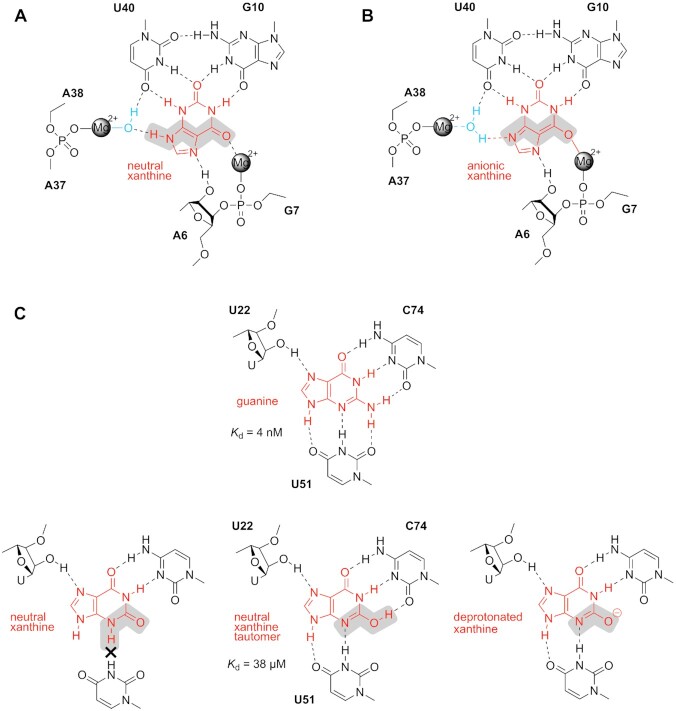
Chemical analysis of xanthine recognition by RNA taking tautomeric and deprotonated ligand forms into account. (**A**) Model for recognition of neutral xanthine by the *NMT1* xanthine riboswitch (this study). (**B**) Model for recognition of deprotonated xanthine by the *NMT1* xanthine riboswitch (this study). (**C**) Models for recognition of guanine *versus* xanthine by the guanine riboswitch based on an earlier study (PDB code: 3GAO) (48). The Mg^2+^-mediated binding mode of the xanthine riboswitch tolerates both the neutral and the deprotonated form of xanthine due to the easily adaptable hydration mode of Mg^2+^ ions that allows for charge compensation.

For a deeper understanding of xanthine recognition, its pH dependence has to be taken into account. The p*K*_a_ of xanthine N3-H is 7.7 and therefore at physiological pH, a significant population of xanthine is deprotonated (monoanion). We therefore analyzed the potential H-bond patterns for neutral as well as deprotonated xanthine in the pocket (Figure 8). It becomes obvious that a tautomeric form of deprotonated xanthine can retain G10-U40 recognition (Figure 8B); thereby, the negative charge at the O6 atom is compensated by the Mg^2+^ ion (M2) that is bound to the phosphate between A6 and G7 (Figure 8B). Simultaneously, the switch from N9-H (neutral xanthine) to the N9 imine (deprotonated xanthine) is compensated by the altered hydration mode of the second Mg^2+^ ion (M3) located at the backbone of A38 (Figure 8A, B). The chemical structure analysis suggests that the binding pocket fulfils the requirements for binding of both neutral and anionic xanthine without an obvious preference for one or the other form. This also suggests that both (neutral and anionic form of xanthine) likely bind with rather comparable affinities. We therefore performed additional ITC experiments at pH 6.0, pH 7.0, and pH 8.0 in the same buffer system (HEPES), and indeed, we found comparable affinities (*K*_d_ (pH 6.0) = 2.6 ± 0.8 μM, *K*_d_ (pH 7.0) = 0.9 ± 0.1 μM, and *K*_d_ (pH 8.0) = 2.2 ± 0.2 μM. (Supplementary Table S2, Supplementary Figure S17).

In this context, we note that 8-azaxanthine has a p*K*_a_ of 4.8 and at physiological pH, the monoanion is dominantly populated (47). Also for this system, the binding pocket of the xanthine riboswitch can in principle tolerate both, the neutral ligand (either the 8*H* or 9*H* tautomer) and the deprotonated form (Supplementary Figure S18). This observation is consistent with its unaltered affinity compared to xanthine.

Finally, we juxtapose xanthine recognition by the xanthine riboswitch to xanthine recognition by the G riboswitch. In an earlier crystallographic study, the G riboswitch was crystalized with a series of guanine analogs including xanthine (48). Although xanthine binds significantly weaker to the G riboswitch (*K*_d_ of 38 μM) compared to its native ligand guanine (*K*_d_ of 4 nM), the recognition pattern by the pocket turned out to be identical. Thereby, the mode of recognition likely involves the N3-imino tautomer or the corresponding deprotonated xanthine (Figure 8C). No metal ion is involved in xanthine recognition by the G riboswitch. This lack of charge compensation in the G riboswitch scaffold compared to the xanthine riboswitch might be the reason for the lower ligand affinity.

Taken together, our study provides deep insights into the molecular principles of ligand recognition of the *NMT1* RNA motif that defines the xanthine riboswitch.

## DATA AVAILABILITY

Atomic coordinates and structure factors for the reported crystal structures have been deposited with the Protein Data bank under accession number 7ELR (xanthine bound), 7ELP (Ir(NH_3_)_6_^3+^-soaked), 7ELQ (Mn^2+^-soaked) and 7ELS (8-azaxanthine bound).

## Supplementary Material

gkab486_Supplemental_File
