# Supplementary material for: Insights into xanthine riboswitch structure and metal ion-mediated ligand recognition
Source: Nucleic Acids Res. 2021 Jun 14;49(12):7139–53. doi: 10.1093/nar/gkab486 (PMC8266621; doi:10.1093/nar/gkab486)
Supplement: gkab486_Supplemental_File [file gkab486_supplemental_file.pdf]

Supporting Materials

Insights into xanthine riboswitch structure and metal ion-mediated ligand recognition

Xiaochen Xu[1,#], Michaela Egger[2,#], Hao Chen[1,#], Karolina Bartosik[2],

Ronald Micura[2,*], and Aiming Ren[1,*]

[1]Life Sciences Institute, Zhejiang University, Hangzhou, Zhejiang 310058, China

[2]Institute of Organic Chemistry, Center for Molecular Biosciences Innsbruck, University of Innsbruck, Innsbruck, 6020, Austria

* To whom correspondence should be addressed. Tel: 86-571-88981227; Fax: 86-571-88981336; Email: aimingren@zju.edu.cn (AR). Correspondence may also be addressed to ronald.micura@uibk.ac.at (RM)

[#]These authors contributed equally.

Contents

Supplementary Figures S1 – S18 2

Supplementary Tables S1 – S2 20

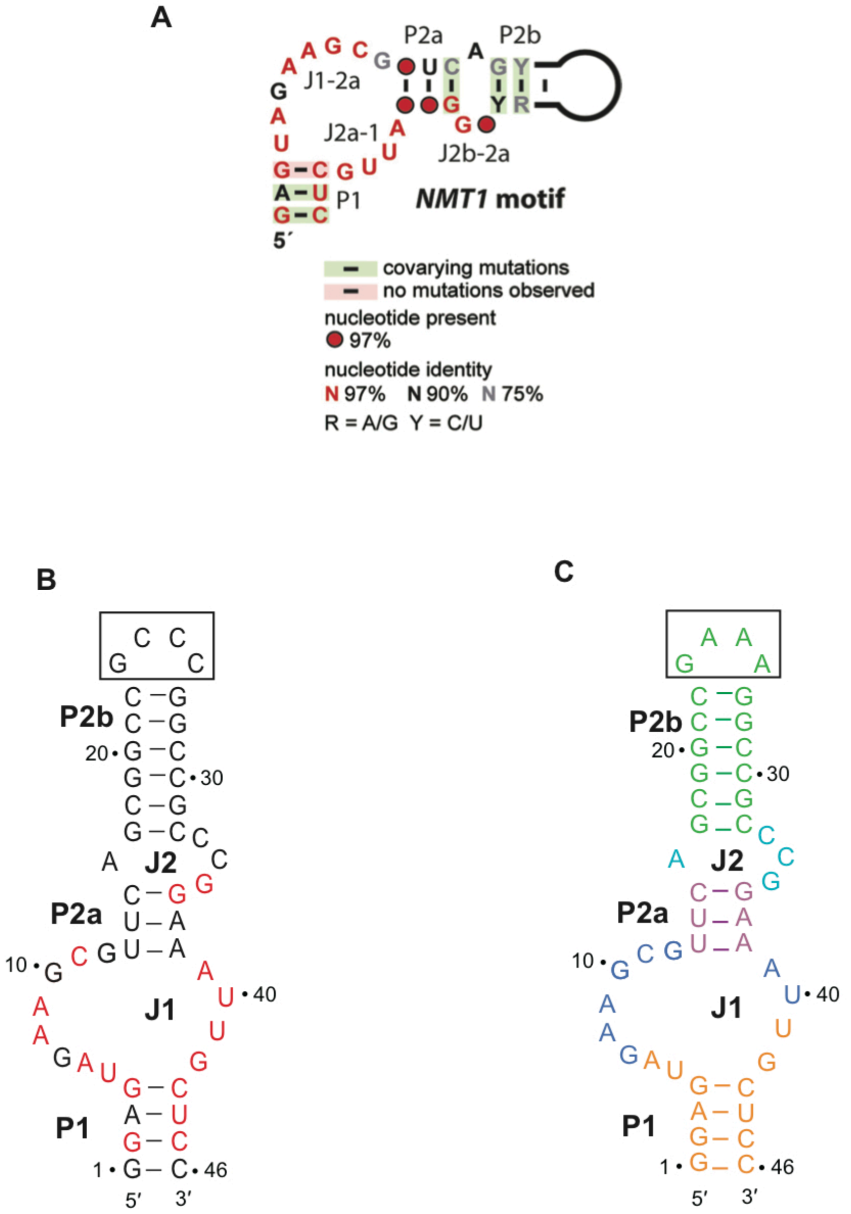

Supplementary Figure S1. *NMT1* riboswitch motif. (**A**) Consensus motif of *NMT1* according to phylogenetic sequence analysis. (**B**) Original RNA sequence used for crystallization trials, with the highly conserved nucleotides shown in red, the variable loop is boxed with a rectangle. (**C**) RNA construct used for crystallographic structure determination. The color of nucleotides refers to stems and junction code in the cartoon representation in Figure 1D.

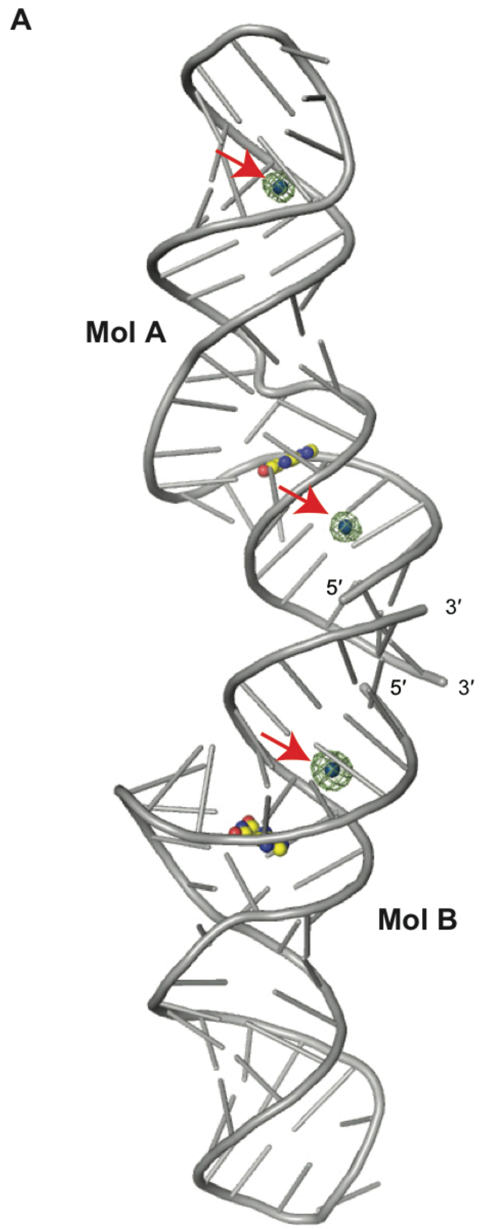

Supplementary Figure S2. Anomalous difference electron density map for $Ir(NH_3)_6^{3+}$-soaked xanthine-bound *NMT1* riboswitch crystals. (**A**) Anomalous electron density map contoured at level 3.0 σ for $Ir(NH_3)_6^{3+}$ sites (deep green balls, labelled with red arrow) in one asymmetric unit of xanthine-bound *NMT1* riboswitch, which were used to solve the phase problem of the structure.

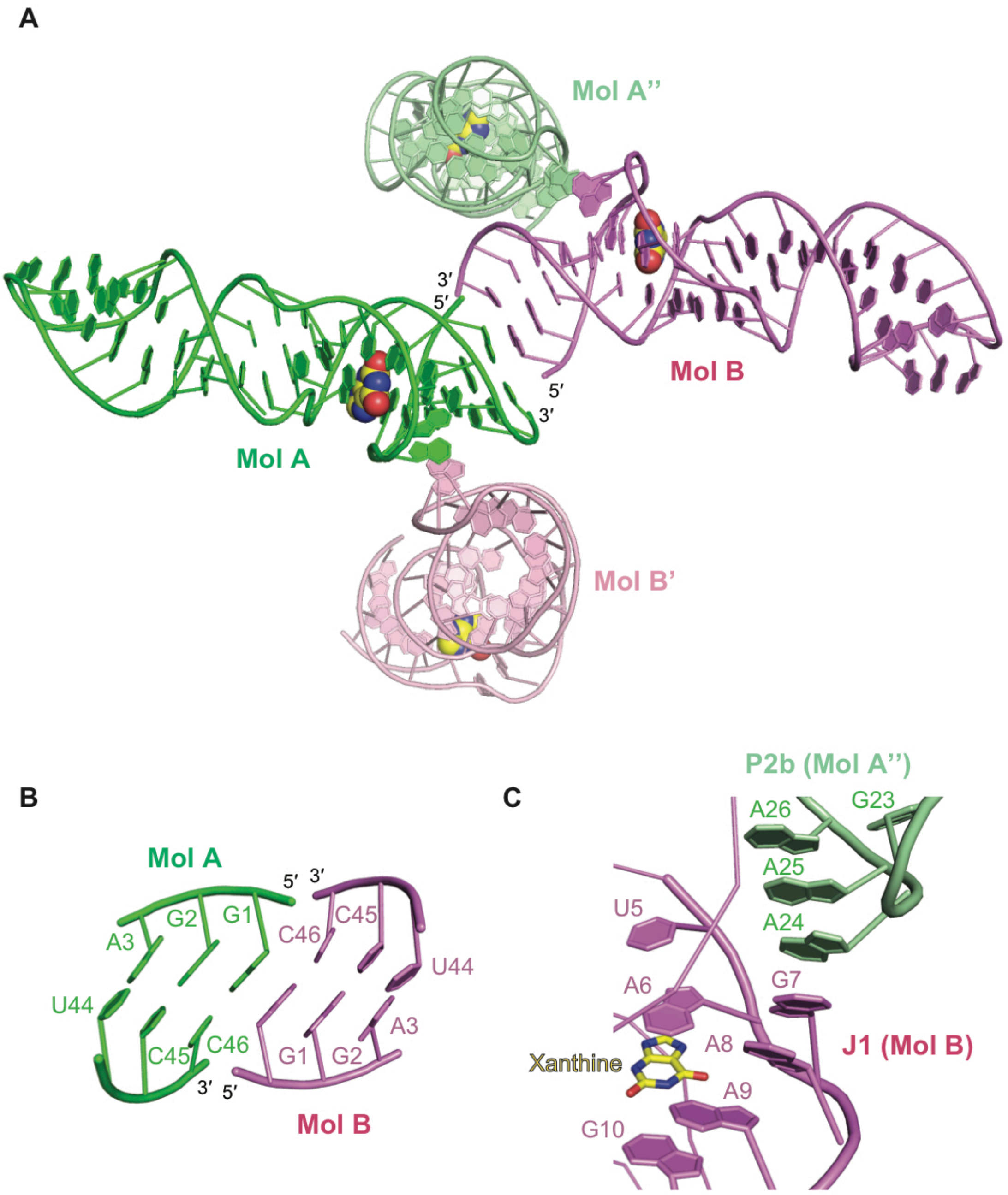

Supplementary Figure S3. Crystal packing interactions of *NMT1* riboswitch molecules. (**A**) One asymmetric unit of *NMT1* riboswitch contains two end-to-end packing RNA molecules, Mol A (green) and Mol B (purple). Both Mol A and Mol B form additional packing interaction with symmetry-related molecule Mol A' (light green) and Mol B'' (pink). (**B**) Mol A (green) and Mol B (purple) adopt end-to-end stacking interaction. (**C**) A close-up view of the packing interaction between Mol B (purple) and symmetry-related molecule Mol A' (light green), in which a continuous purine stacking pattern formed with A26-A25-A24 from Mol A' (light green) and G7-A8-A9-G10 from Mol B (purple).

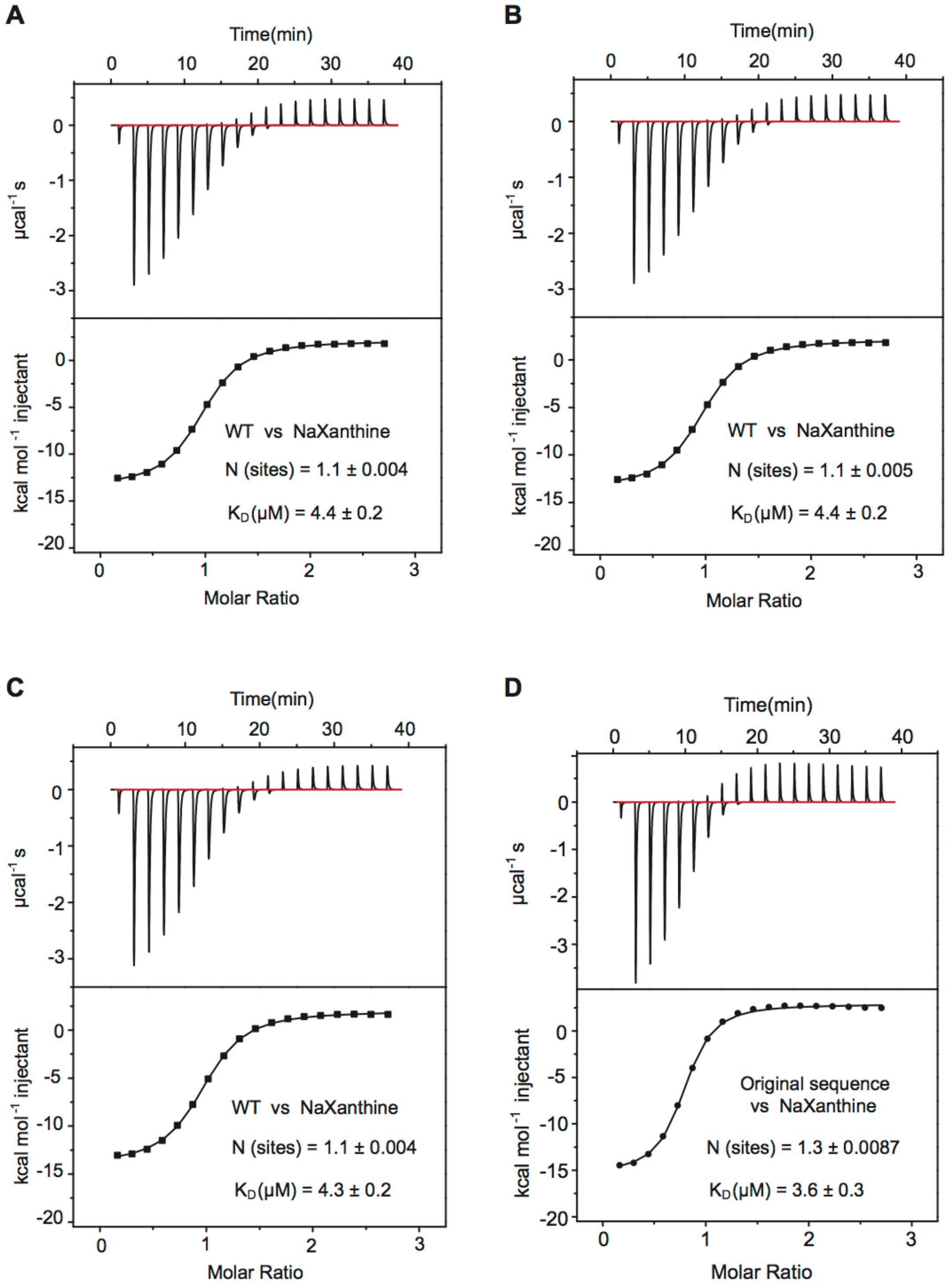

Supplementary Figure S4. Isothermal titration calorimetry (ITC) of *NMT1* riboswitch bound to xanthine. (**A-C**) Three independent ITC titration replicates of the wild-type (WT) xanthine riboswitch sequence used in crystallization (GAAA loop). (**D**) ITC titration of the original sequence of xanthine riboswitch (GCCC loop, see Supplementary Figure 1B). All titrations were performed at 25 °C in 50 mM Tris pH 8.0, 50 mM KCl and 10 mM $MgCl_2$. For c-values see Supplementary Table S2. The K_d and N parameters obtained from the fits are shown as text in the individual ΔH windows.

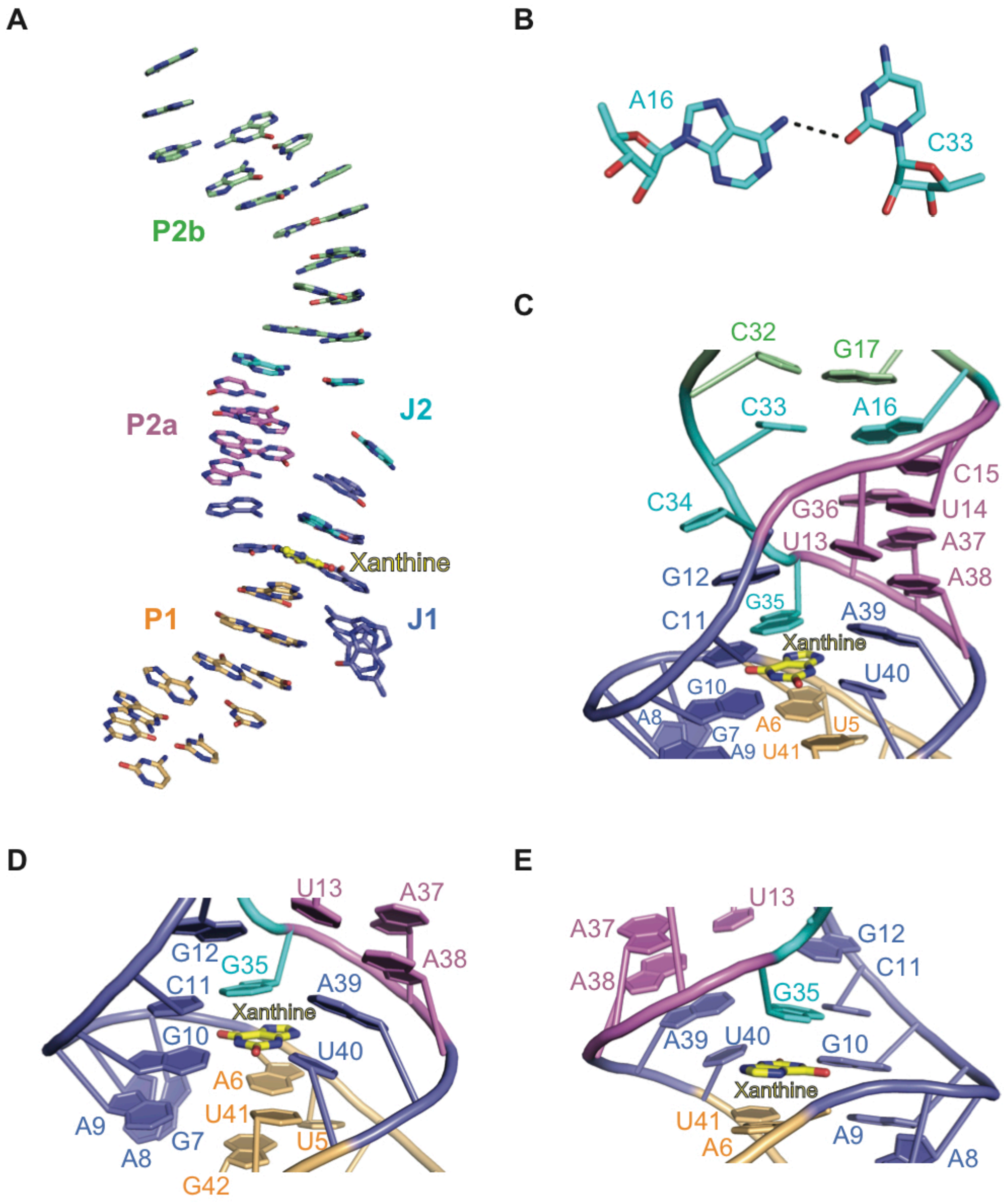

Supplementary Figure S5. Structural details of *NMT1* riboswitch bound to xanthine. (**A**) Base stacking alignment of *NMT1* riboswitch and the bound xanthine. (**B**) A16 and C33 form one wobble base pair between stems P2a and P2b, in which 6-NH2 of A16 hydrogen bond with O2 of C33. (**C**) A close-up view of the junction interaction between J1 and J2. (**D**) A close-up view of junction J1, in which A39 and U40 form stacking interaction and connect stem P2a and P1 to constitute the long helical fold. (**E**) Xanthine is bracketed by the two strands of J1 and stacked on the top of stem P1.

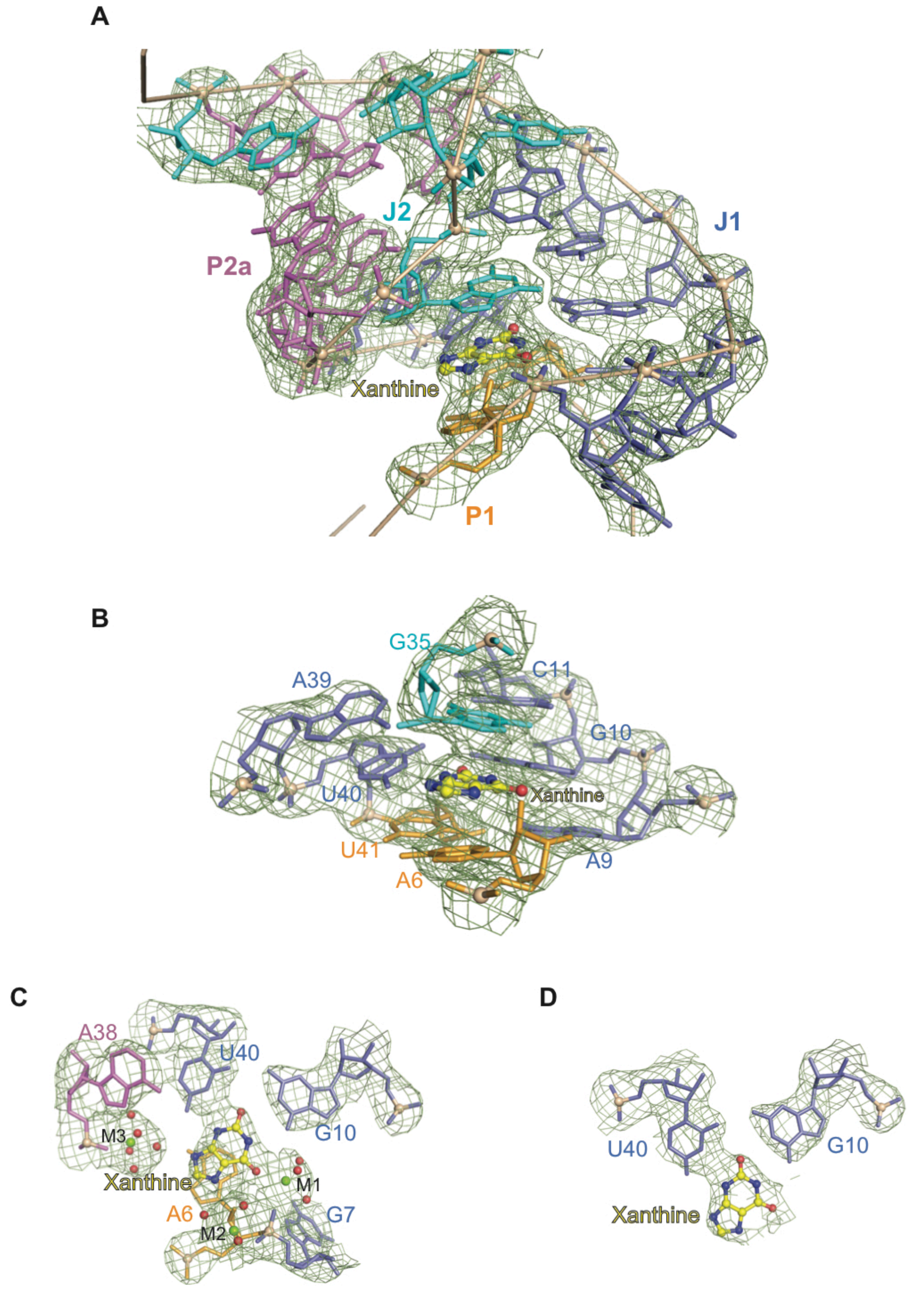

Supplementary Figure S6. $2F_o$-F_c electron density map of the *NMT1* riboswitch bound to xanthine. $2F_o$-F_c electron density map contoured at level 1.0 σ is shown in light gray for the J1-J2 junction region (**A**), the ligand-binding pocket composition and three involved metals ions M2-M4 (**B-C**), and the bound xanthine and two directly interacted residues G10 and U40 (**D**).

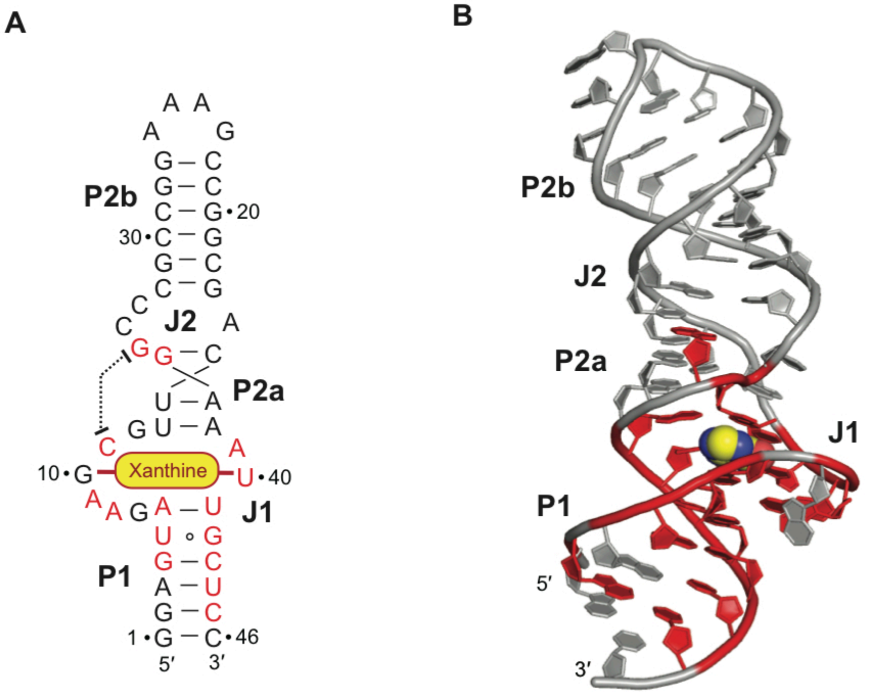

Supplementary Figure S7. Structural alignment of the highly conserved residues in *NMT1* riboswitch. Highly conserved residues are brought into proximity around the binding pocket shown in the secondary structure (**A**) and in the tertiary structure fold (**B**).

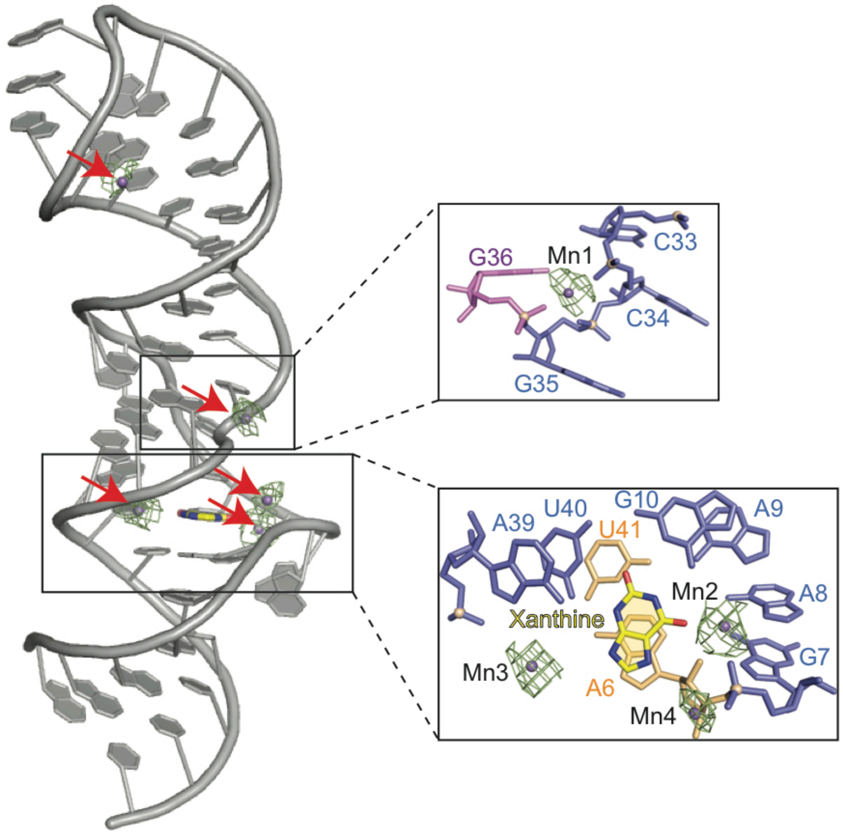

Supplementary Figure S8. Anomalous difference electron density map for Mn^{2+}-soaked *NMT1* riboswitch bound with xanthine. Anomalous electron density map contoured at level 3.0 σ for Mn^{2+} sites (purple balls) of xanthine-bound *NMT1* riboswitch and close-up views of the four cations binding sites including one metal Mn1 (M1) coordinated to the junction region of J2 and three metals Mn2 (M2), Mn3 (M3) and Mn4 (M4) located in the binding pocket of *NMT1* riboswitch.

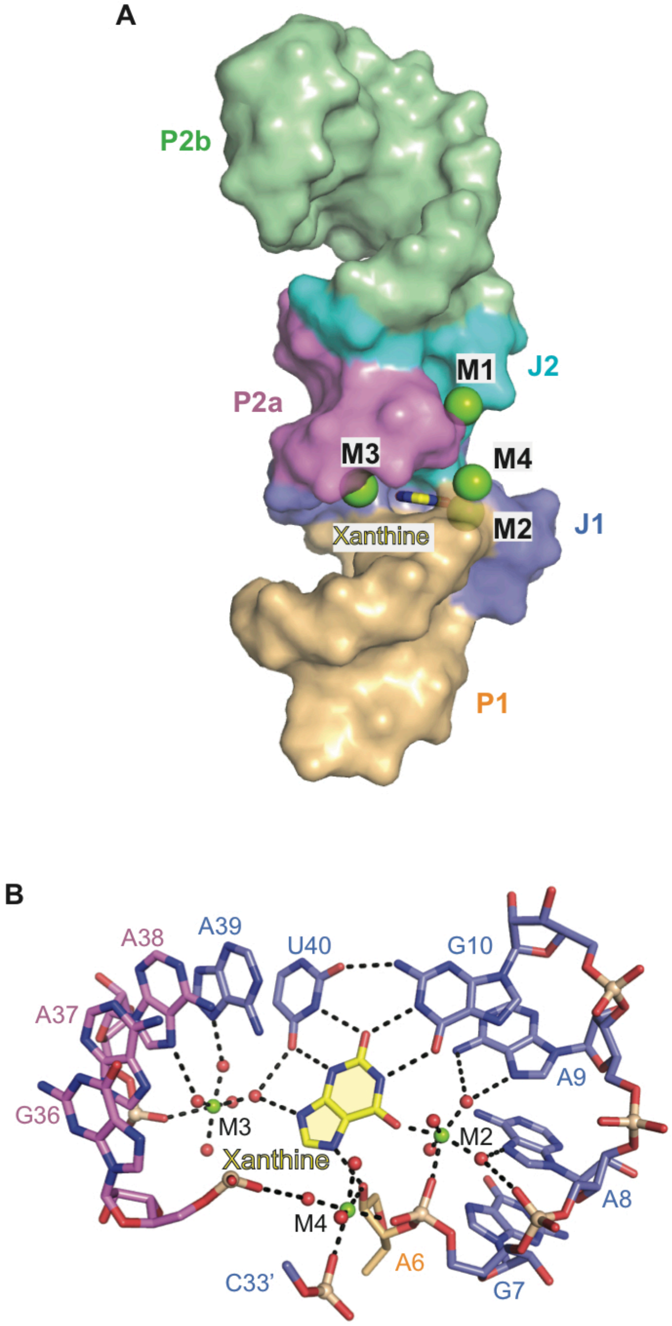

Supplementary Figure S9. Surface representation and the binding pocket interaction of *NMT1* riboswitch bound to xanthine. (**A**) Surface representation of *NMT1* riboswitch bound to xanthine. Xanthine intercalates into the junction region between stem P1 and P2a. Three metals M2, M3 and M4 were identified in the binding pocket. (**B**) The binding pocket interaction of *NMT1* riboswitch bound to xanthine. Xanthine is encapsulated by G10, U40, A6 and two metals M2 and M3, which are further bracketed by two continuous stacking purine chains A9-A8-G7 below G10 and A39-A38-A37-G36 above U40. It is notable that the phosphates and the Hoogsteen edges of these purine residues are also involved in the coordination of metals M2, M3 and M4.

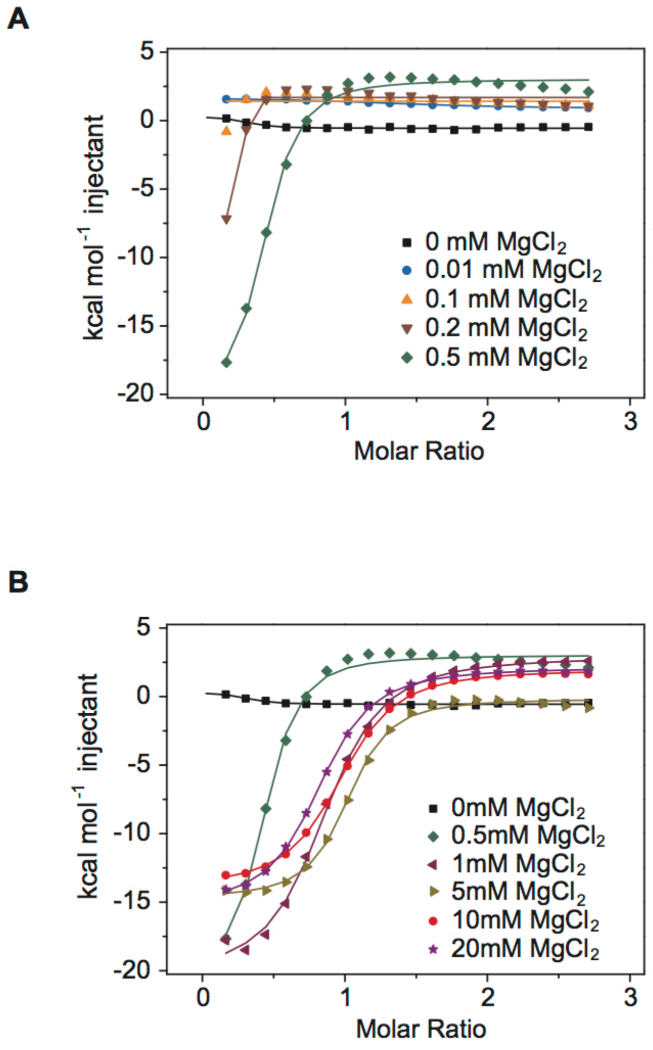

Supplementary Figure S10. Mg^{2+}-dependence of *NMT1* riboswitch binding to xanthine analyzed by ITC. (**A**) Overlay of integrated fitted heat plots obtained from ITC experiments of *NMT1* riboswitch in the presence of xanthine (0.1 mM) at different Mg^{2+} concentration below 0.5 mM (as indicated). (**B**) Overlay of integrated fitted heat plots obtained from ITC experiments of *NMT1* riboswitch in the presence of xanthine (0.1 mM) at different Mg^{2+} concentration ranging from 0.5 mM to 20 mM (as indicated). All titrations were performed at 25 °C in 50 mM Tris pH 8.0, 50 mM KCl, supplemented with different concentration of $MgCl_2$. For K_d, N parameters and c-values see Supplementary Table S2.

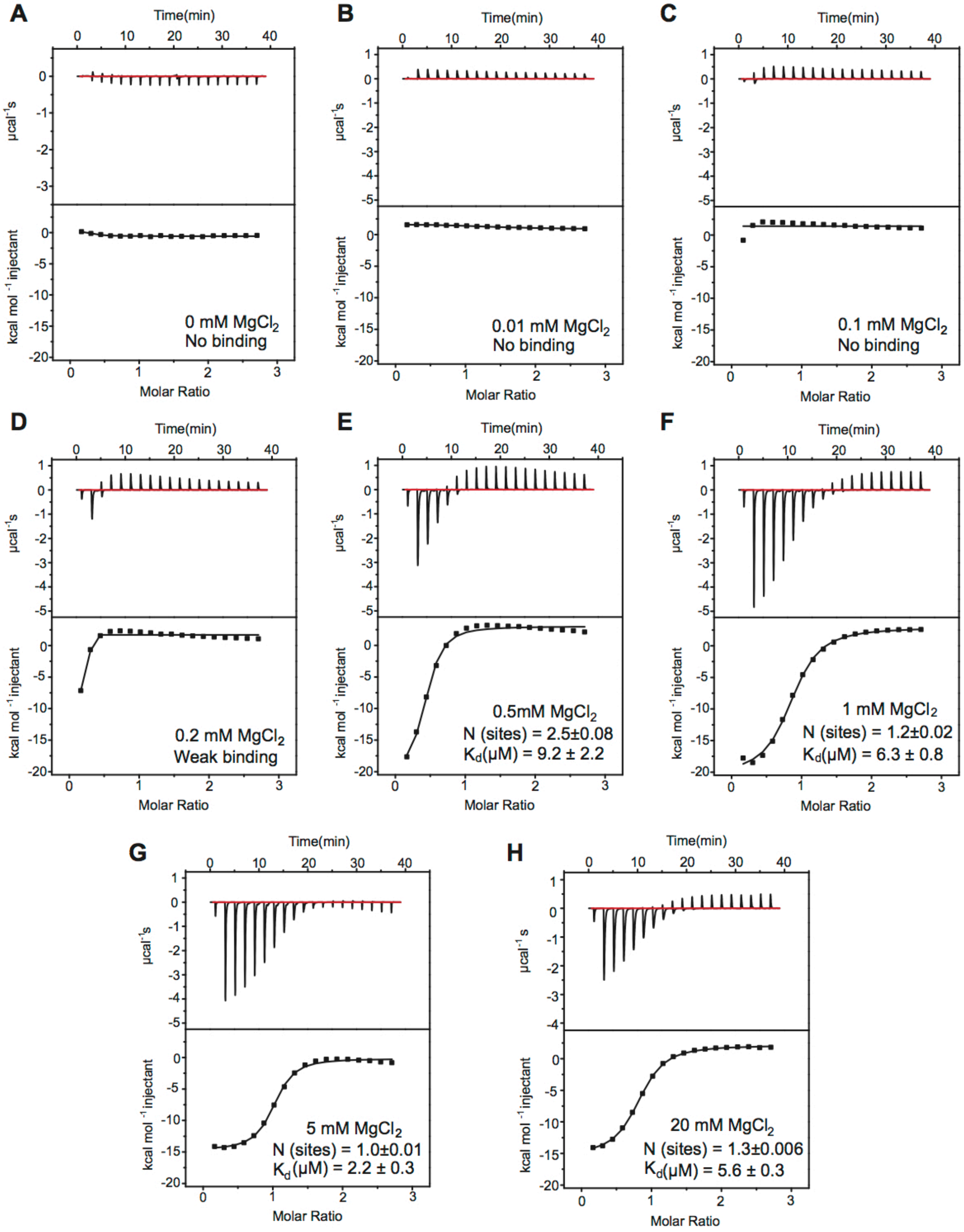

Supplementary Figure S11. Selected ITC experiments to analyze Mg^{2+}-dependence of *NMT1* riboswitch-xanthine binding. ITC plots in the presence of a Mg^{2+} concentration of 0 mM (**A**), 0.01 mM (**B**), 0.1 mM (**C**), 0.2 mM (**D**), 0.5 mM (**E**), 1 mM (**F**), 5 mM (**G**) and 20 mM (**H**). All titrations were performed at 25 °C in 50 mM Tris pH 8.0, 50 mM KCl, supplemented with different concentration of $MgCl_2$. For c-values see Supplementary Table S2. The K_d and N parameters obtained from the fits are shown as text in the individual ΔH windows.

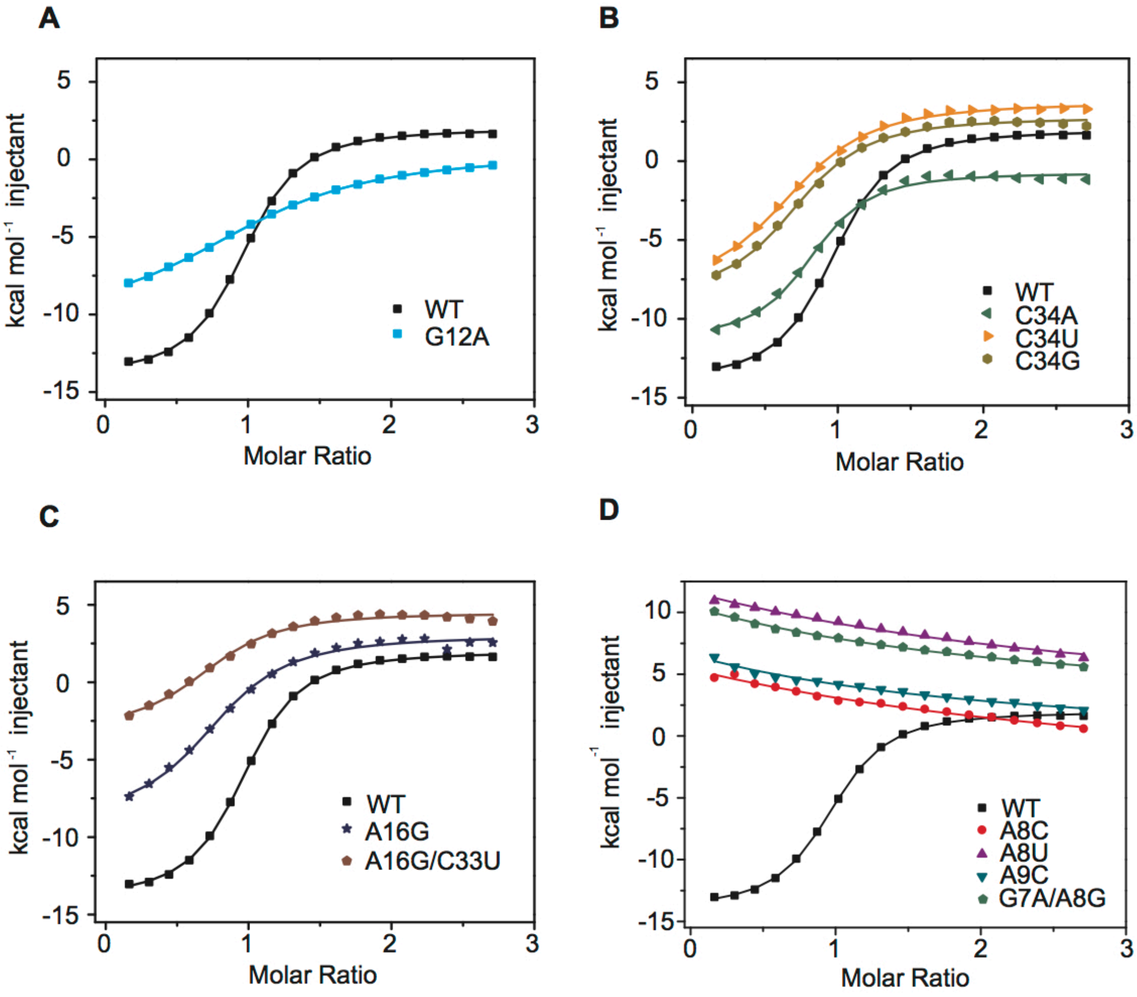

Supplementary Figure S12. Selected ITC experiments to analyze xanthine binding to *NMT1* riboswitch and mutants. (**A**) Overlay of integrated fitted heat plots obtained from ITC experiments of *NMT1* riboswitch and mutant G12A binding to xanthine. G12 forms base-specific interaction with the phosphate of G35. (**B**) Overlay of integrated fitted heat plots obtained from ITC experiments of *NMT1* riboswitch and mutant of C34 binding to xanthine. C34 forms a base-specific hydrogen bond with the sugar of G12. (**C**) Overlay of integrated fitted heat plots obtained from ITC experiments of *NMT1* riboswitch and A16G, A16G/C33U mutants. (**D**) Overlay of integrated fitted heat plots obtained from ITC experiments of *NMT1* riboswitch and A7, A8 mutants binding to xanthine. A7 and A8 from J1 form hydrogen bond interactions with second terminal base pair G42-U5 of stem P1.

A

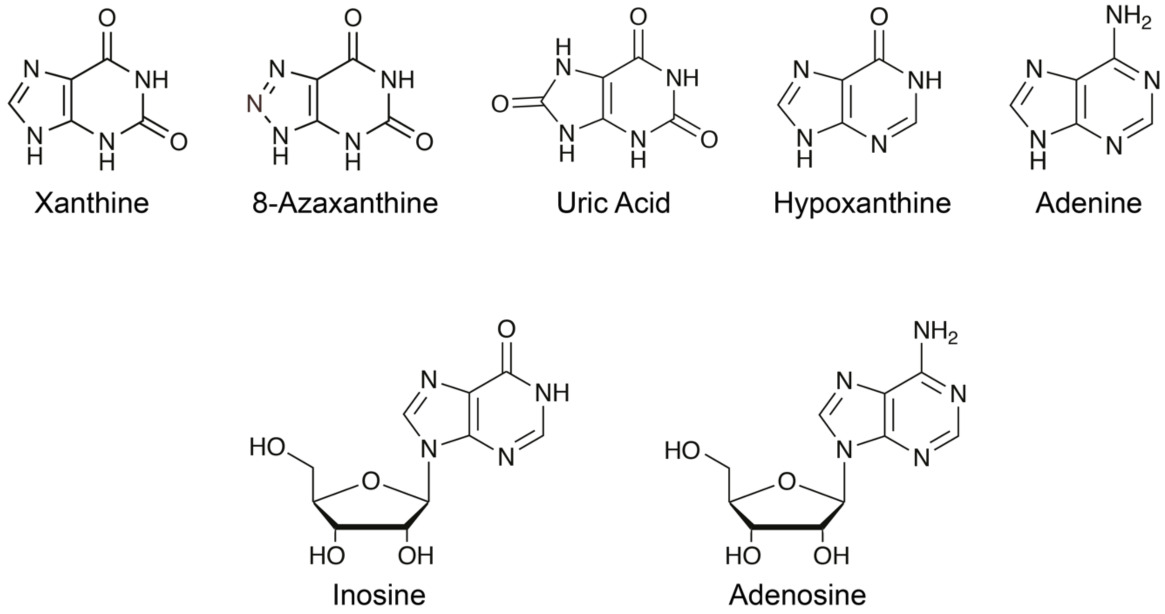

B

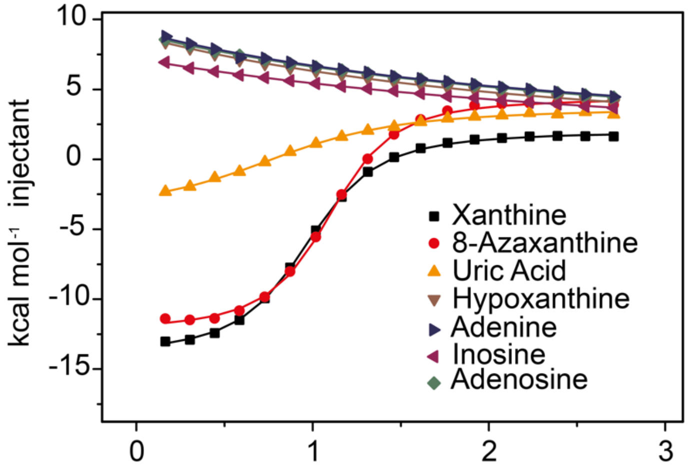

Supplementary Figure S13. ITC experiments to analyze *NMT1* riboswitch binding to xanthine analogs. (**A**) The chemical structure of xanthine, 8-azaxanthine, uric acid, hypoxanthine, adenine, inosine and adenosine. (**B**) Overlay of integrated fitted heat plots obtained from ITC experiments of *NMT1* riboswitch binding to xanthine and analogs as shown in (A). All titrations were performed at 25 °C in 50 mM Tris pH 8.0, 50 mM KCl, and 10 mM $MgCl_2$. For K_d, N parameters and c-values see Supplementary Table S2.

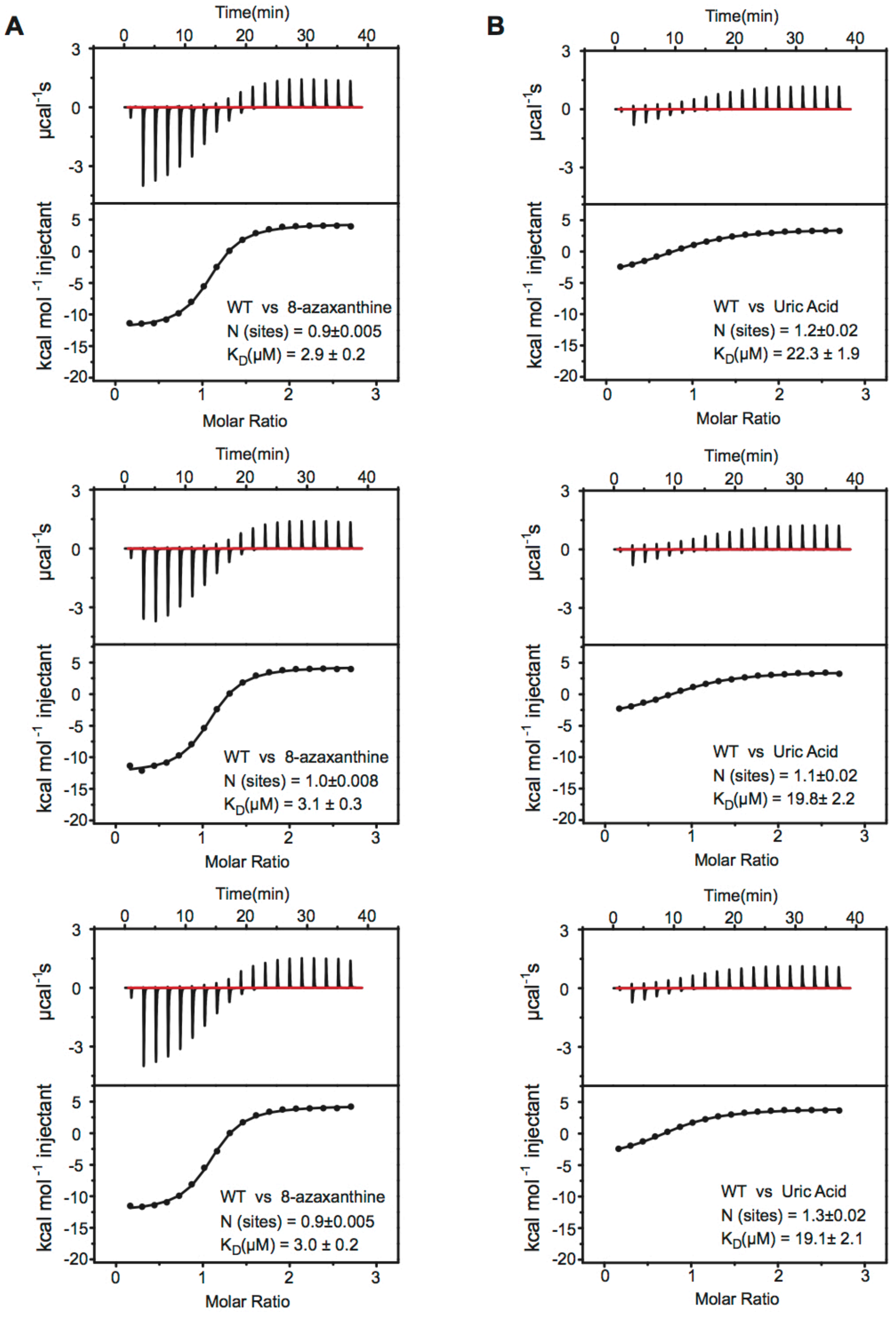

Supplementary Figure S14. Independently repeated ITC experiments of *NMT1* riboswitch bound to 8-azaxathine and uric acid. Independently repeated ITC binding curves of *NMT1* riboswitch bound to 8-azaxathine (**A**) and uric acid (**B**). All titrations were performed at 25 °C in 50 mM Tris pH 8.0, 50 mM KCl, and 10 mM $MgCl_2$. For K_d, N parameters and c-values see Supplementary Table S2.

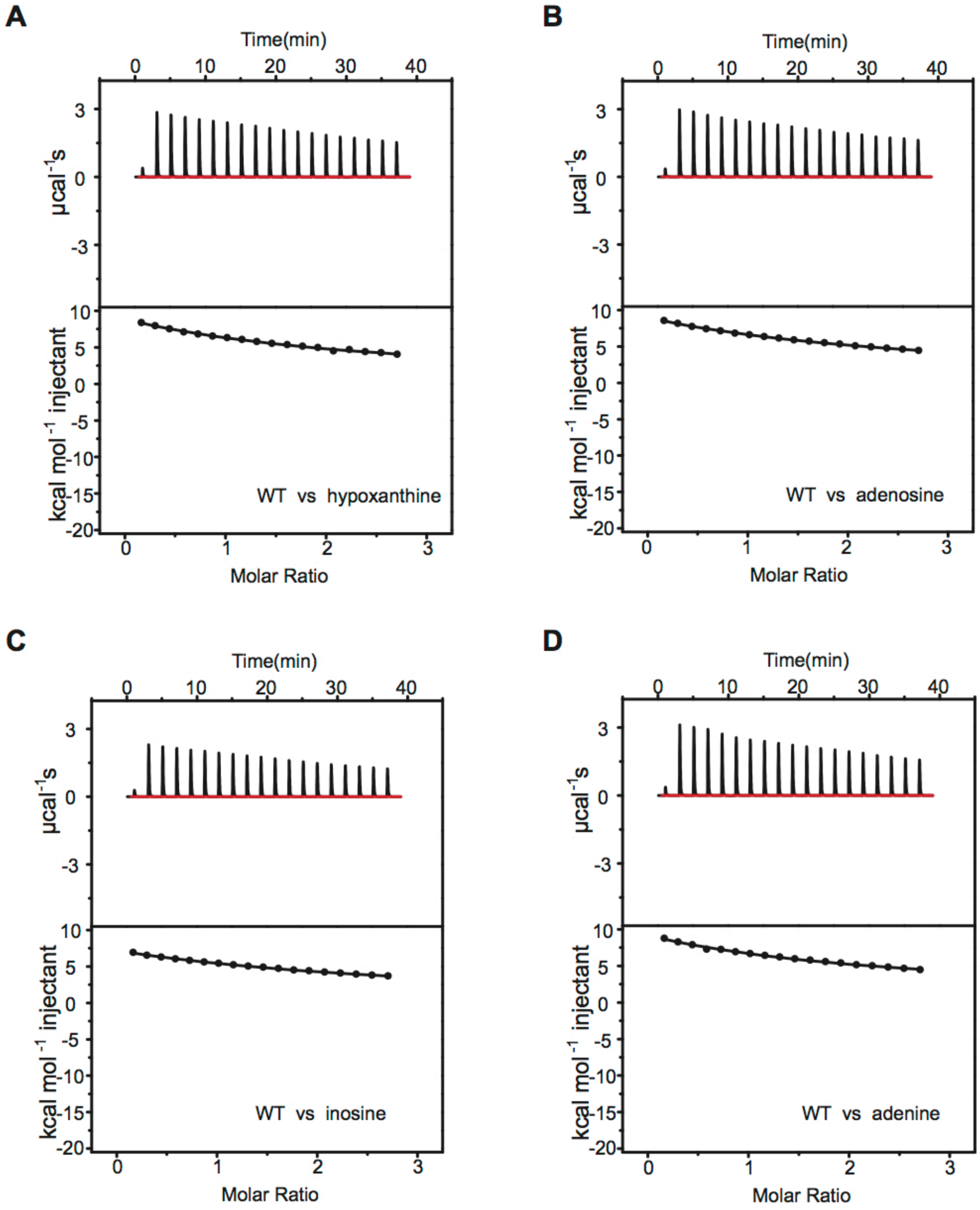

Supplementary Figure S15. ITC binding curves of *NMT1* riboswitch bound to some xanthine analogs. ITC binding curves of *NMT1* riboswitch bound to hypoxanthine (**A**), adenine (**B**), inosine (**C**) and adenosine (**D**). All titrations were performed at 25 °C in 50 mM Tris pH 8.0, 50 mM KCl, and 10 mM $MgCl_2$. For K_d, N parameters and c-values see Supplementary Table S2.

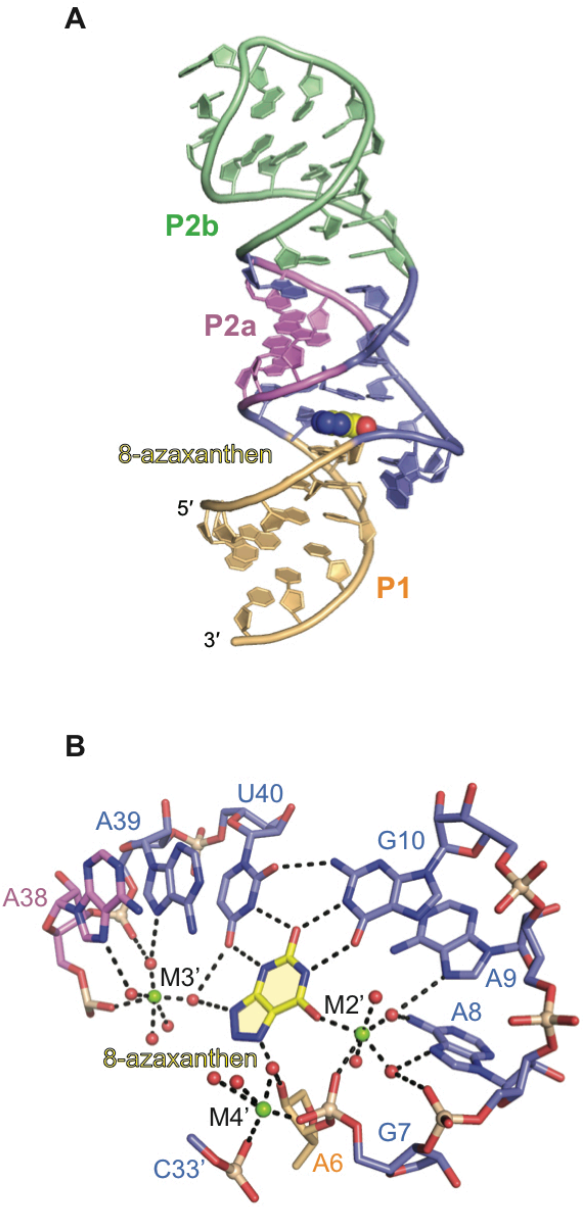

Supplementary Figure S16. Structure of *NMT1* riboswitch in complex with 8-azaxanthine. (**A**) Overall tertiary structure of *NMT1* riboswitch (shown in cartoon representation) bound with 8-azaxanthine, in which the colors are coded to Figure 1D. (**B**) The interaction of the 8-azaxanthine binding pocket of NMT1 riboswitch are similar with xanthine binding pocket. For the detail, please see the illustration of Supplementary Figure S9B.

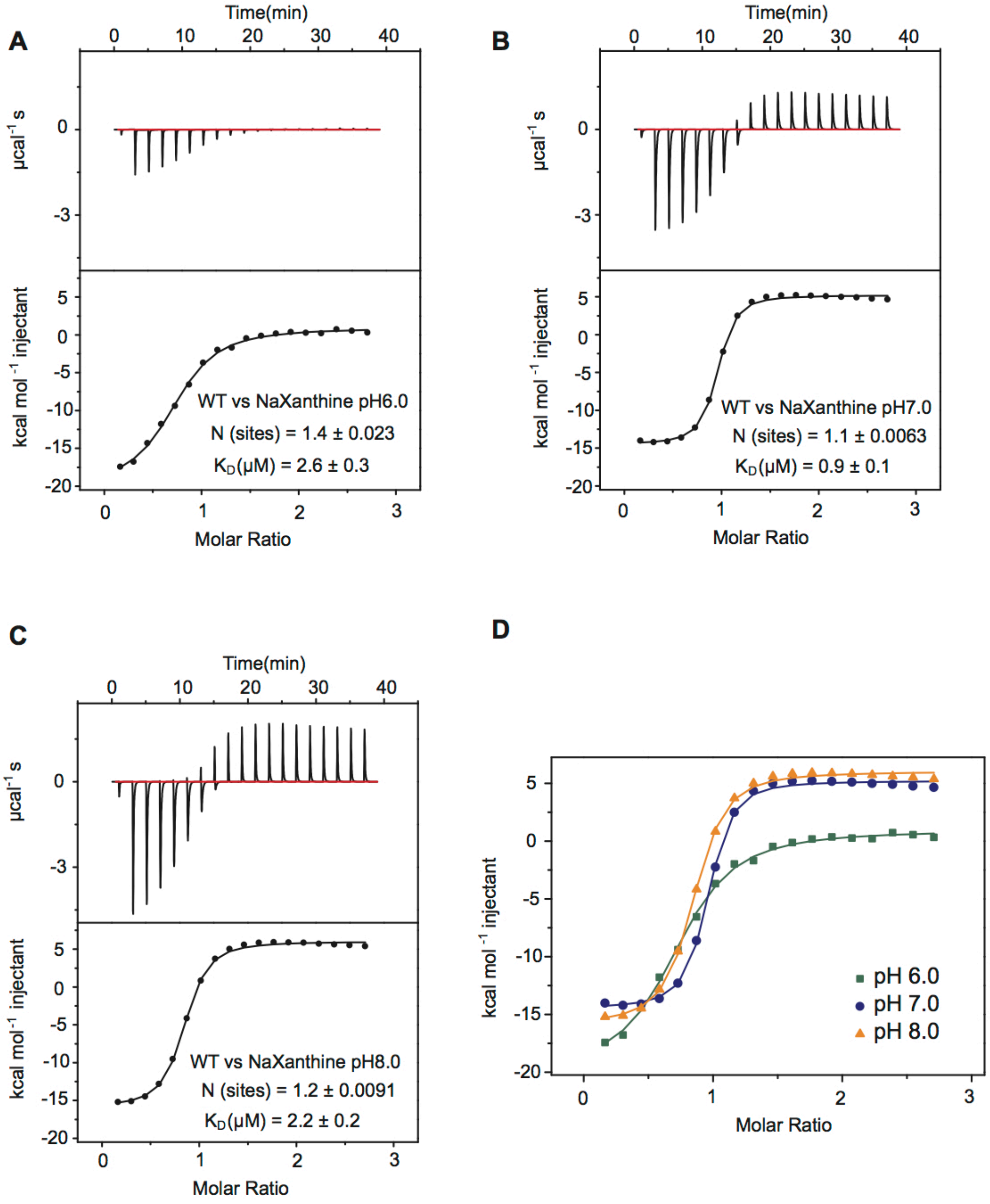

Supplementary Figure S17. The pH-dependence ITC experiments of *NMT1* riboswitch binding to xanthine. ITC binding curves of *NMT1* riboswitch bound to xanthine at pH 6.0 (**A**), pH 7.0 (**B**) and pH 8.0 (**C**). (**D**) Overlay of integrated fitted heat plots obtained from pH dependence ITC experiments of *NMT1* riboswitch binding to xanthine. All titrations were performed at 25 °C in 50 mM HEPES (different pH), 50 mM KCl, and 10 mM $MgCl_2$. For K_d, N parameters and c-values see Supplementary Table S2. Due to the low solubility of xanthine at pH 6.0, the concentration of RNA and ligand used in the titration of pH 6.0 was reduced by 4-fold to 0.35 mM RNA and 25 µM xanthine.

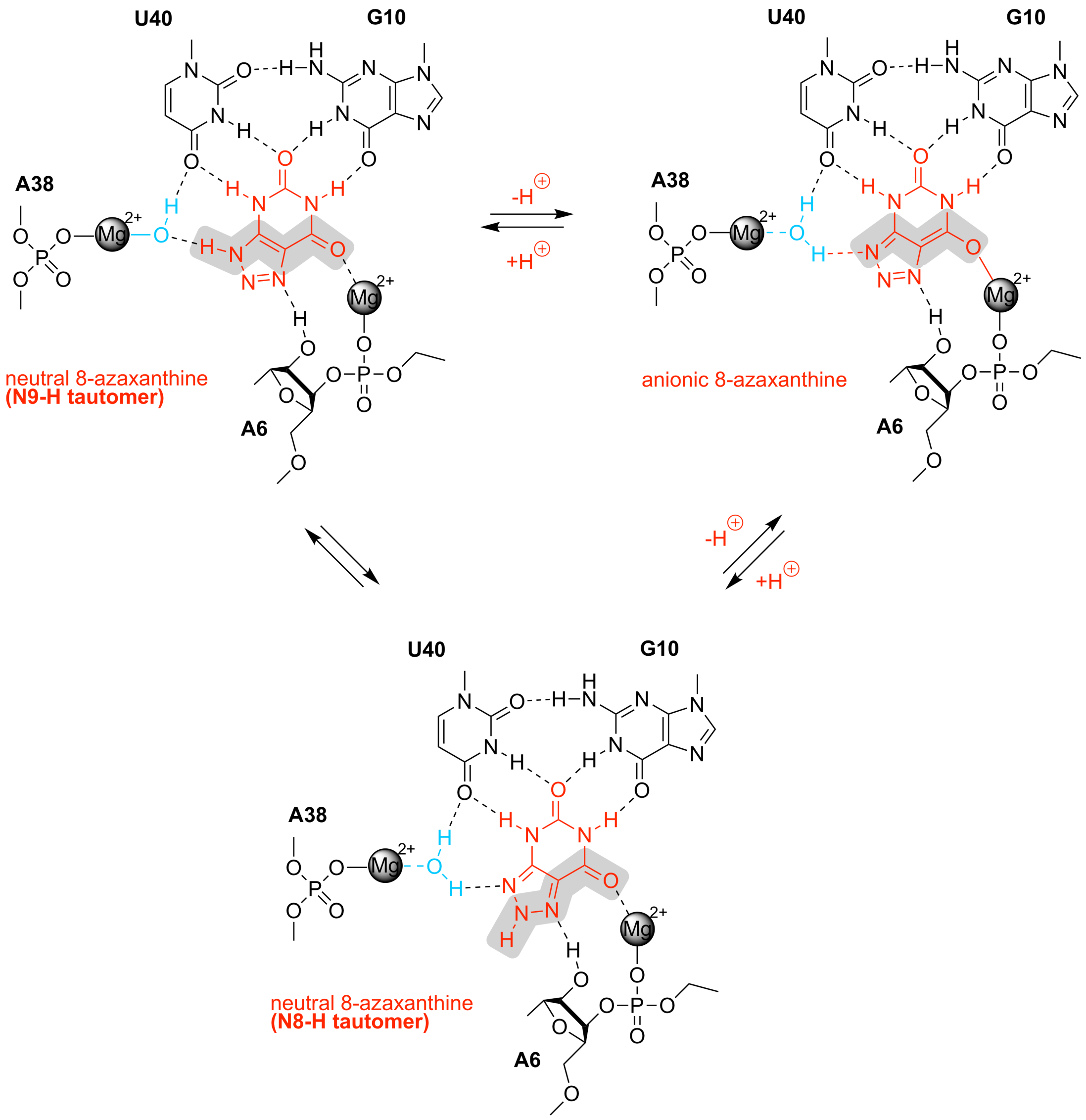

Supplementary Figure S18. Chemical analysis of ligand recognition of the *NMT1* riboswitch in complex with 8-azaxanthine. Chemical structure analysis considering the two dominant tautomeric forms of 8-azaxanthine and the corresponding deprotonated ligand. The Mg^{2+}-mediated binding tolerates both the neutral and the deprotonated form of 8-azaxanthine due to the easily adaptable hydration mode of the Mg^{2+} ions, that allows for charge compensation.

Supplementary Table S1. Crystallographic statistics of xanthine riboswitches.

NMT1 riboswitch	Xanthine-bound	$Ir(NH_3)_6^{3+}$-soaked	Mn^{2+}-soaked	8-azaxanthine -bound
**Data collection**				
Space group	$P\ 2_12_12$	$P\ 2_12_12$	$P\ 2_12_12$	$P\ 2_12_12$
a, b, c (Å)	80.4, 96.2, 41.5	79.5, 100.2, 40.3	79.5, 92.9, 40.3	79.7, 93.1, 40.1
α, β, ϒ (º)	90,90,90	90,90,90	90,90,90	90,90,90
Wavelength(Å)	1.102	1.102	1.2398	1.102
Resolution (Å)	50.0-2.66(2.76-2.66)	50.0-2.8(2.9-2.8)	50.0-2.6(2.7-2.6)	50.0-2.8(2.9-2.8)
R_{pim}	0.03(0.3)	0.04(0.4)	0.04(0.3)	0.04(0.4)
$I/\delta I$	30.8(1.3)	29.8(1.3)	20.0(1.3)	27.4(1.0)
Completeness (%)	98.0 (84.9)	99.8 (99.2)	80.3 (34.6)	98.6 (89.0)
Redundancy	12.0 (9.6)	12.5 (11.6)	10.4 (5.0)	11.7 (9.0)
$CC_{1/2}$	1.0 (0.9)	0.9(0.7)	1.0(0.5)	1.0(0.8)
**Refinement**				
Resolution (Å)	48.1-2.66(2.76-2.66)	39.7-2.8(2.9-2.8)	46.5-2.6(2.7-2.6)	40.0-3.0(3.1-3.0)
No.reflections	9427	8418	7759	6307
R_{work}/R_{free} (%)	24.3/26.5	21.5/27.4	21.1/26.1	23.2/27.4
No. of atoms				
RNA	1998	1998	1998	1998
Ligand	22	22	22	22
Mg^{2+}	15	16	9	13
Water	54	85	80	46
Mn^{2+}			8	
Ir^{-}		3		
B-factors($Å^2$)				
RNA	100.3	84.8	55.8	84.9
Ligand	93.0	74.8	49.8	75.6
Mg^{2+}	93.4	80.1	57.4	80.1
Water	94.3	82.6	52.3	78.7
Mn^{2+}			60.1	
Ir^{-}		113.6		
R.m.s deviations				
Bond lengths (Å)	0.002	0.006	0.005	0.003
Bond angles (º)	0.7	1.2	1.1	0.8

*Values for the highest-resolution shell are in parentheses.

Supplementary Table S2. Thermodynamic parameters of ligand binding to xanthine riboswitch determined by isothermal titration calorimetry (ITC).

RNA[a]	Ligand	ΔH	-TΔS	ΔG	ΔΔG	$N^{(b)}$	$K_d^{(c)}$ [μM]	c-value	$K_d^{(d)}$ (Mean)
			[kcal/mol]		[kcal/mol]				[μM]
WT<br>(10mM $MgCl_2$)	Na Xanthine	-14.7±0.1<br>-14.8±0.1<br>-15.1±0.1	7.4<br>7.5<br>7.8	-7.3<br>-7.3<br>-7.3	--	1.1±4.1e^{-3}<br>1.1±4.5e^{-3}<br>1.1±3.8e^{-3}	4.3±0.2<br>4.4±0.2<br>4.4±0.2	23.3<br>22.7<br>22.7	4.4±0.1
WT<br>(10mM $MgCl_2$)	8-azaxanthine	-17.7±0.2<br>-17.8±0.3<br>-17.9±0.2	10.1<br>10.2<br>10.4	-7.6<br>-7.5<br>-7.5	-0.2	0.9±5.3e^{-3}<br>1.0±7.5e^{-3}<br>0.9±5.3e^{-3}	2.9±0.2<br>3.1±0.3<br>3.0±0.2	34.5<br>32.3<br>33.3	3.0±0.1
WT<br>(10mM $MgCl_2$)	Uric acid	-6.8±0.2<br>-6.5±0.2<br>-6.2±0.2	0.4<br>0.1<br>-0.2	-6.4<br>-6.4<br>-6.4	0.9	1.2±1.5e^{-2}<br>1.1±1.9e^{-2}<br>1.3±2.1e^{-2}	22.3±1.9<br>19.8±2.2<br>19.1±2.1	4.5<br>5.1<br>5.2	20.4±1.7
WT<br>(10mM $MgCl_2$)	Hypoxanthine						N.D.[e]		
WT<br>(10mM $MgCl_2$)	Adenosine						N.D.		
WT<br>(10mM $MgCl_2$)	Adenine						N.D.		
WT<br>(10mM $MgCl_2$)	Inosine						N.D.		
WT<br>(0mM $MgCl_2$)	Na Xanthine						N.D.		
WT<br>(0.01mM $MgCl_2$)	Na Xanthine						N.D.		
WT<br>(0.1mM $MgCl_2$)	Na Xanthine						N.D.		
WT<br>(0.2mM $MgCl_2$)	Na Xanthine						N.D.		
WT<br>(0.5mM $MgCl_2$)	Na Xanthine	-9.5±0.4	2.6	-6.9	0.4	2.48±7.9e^{-2}	9.2±2.2	10.9	
WT<br>(1mM $MgCl_2$)	Na Xanthine	-19.6±0.5	12.5	-7.1	0.2	1.2±1.6e^{-2}	6.3±0.8	15.9	
WT<br>(5mM $MgCl_2$)	Na Xanthine	-14.2±0.3	6.5	-7.7	-0.4	1.0±1.1e^{-2}	2.2±0.3	45.5	
WT<br>(20mM $MgCl_2$)	Na Xanthine	-14.0±0.1	6.8	-7.2	0.1	1.3±6.2e^{-3}	5.6±0.3	17.9	

WT (pH 6.0)	Na Xanthine	-15.2±0.4	7.5	-7.6	-0.3	1.4±2.3e^{-2}	2.6±0.3	38.5	
WT (pH 7.0)	Na Xanthine	-17.8±0.2	9.5	-8.2	-0.9	1.1±6.3e^{-3}	0.9±0.1	111.1	
WT (pH 8.0)	Na Xanthine	-17.6±0.2	9.8	-7.7	-0.4	1.2±9.1e^{-3}	2.2±0.2	45.5	
Original sequence	Na Xanthine	-13.7±0.2	6.3	-7.4	-0.1	1.3±8.7e^{-3}	3.6±0.3	27.8	
A8C	Na Xanthine						N.D.		
A8U	Na Xanthine						N.D.		
G7A/A8G	Na Xanthine						N.D.		
A9C	Na Xanthine						N.D.		
G10A	Na Xanthine						N.D.		
G10C	Na Xanthine						N.D.		
U40A	Na Xanthine						N.D.		
U40C	Na Xanthine						N.D.		
G10CU40A	Na Xanthine						N.D.		
C11G	Na Xanthine						N.D.		
G12A	Na Xanthine	-11.8 ± 0.2	5.5	-6.3	1.0	0.9±5.6e^{-3}	25.9±1.0	3.9	
G35C	Na Xanthine						N.D.		
C11U/G35A	Na Xanthine						N.D.		
C11G/G35C	Na Xanthine						N.D.		
A16G	Na Xanthine	-9.3±0.4	2.6	-6.7	0.6	1.3±3.1e^{-2}	13.0±2.3	7.7	
A16GC33U	Na Xanthine	-5.6±0.3	-1.0	-6.7	0.6	1.4±4.7e^{-2}	13.4±3.3	7.5	
C34G	Na Xanthine	-8.4±0.3	1.7	-6.7	0.6	1.4±2.7e^{-2}	12.1±1.8	8.3	
C34A	Na Xanthine	-8.6±0.3	1.5	-7.1	0.2	1.2±2.3e^{-2}	6.3±1.1	15.9	
C34U	Na Xanthine	-8.7±0.3	2.2	-6.5	0.8	1.4±2.4e^{-2}	17.0±2.0	5.9	

[a] WT refers to the sequence shown in Supplementary Figure 1C (GAAA loop).

[b] N, the stoichiometric ratio of ligand to RNA.

[c] The binding disassociation parameter fitted for each independent titration.

[d] Reported error is the standard deviation of three independent experiments.

[e] N.D. refers to no detectable interaction under the experiment conditions.
